# Trends in all-cause and cause-specific mortality by BMI levels in England, 2004–2019: a population-based primary care records study

**DOI:** 10.1016/j.lanepe.2024.100986

**Published:** 2024-07-02

**Authors:** Marisa K. Sophiea, Francesco Zaccardi, Yiling J. Cheng, Eszter P. Vamos, Naomi Holman, Edward W. Gregg

**Affiliations:** a Department of Epidemiology and Biostatistics, School of Public Health, Imperial College London, White City Campus, London W12 7TA, UK; b Leicester Real World Evidence Unit, Leicester Diabetes Centre, University of Leicester, Leicester General Hospital, Leicester LE5 4PW, UK; c US Centers for Disease Control and Prevention, 4770 Buford Highway NE, MS S107-3, Atlanta, GA, 30341, USA; d Department of Primary Care and Public Health, School of Public Health, Imperial College London, Charing Cross Campus, London W6 8RP, UK; e School of Population Health, RCSI University of Medicine and Health Sciences, Beaux Lane House, Lower Mercer Street, Dublin 2, Ireland

**Keywords:** Body mass index, All-cause mortality, Cardiovascular disease, Proportional mortality, Trends

## Abstract

**Background:**

In the UK, obesity rates are rising concurrently with declining mortality rates. Yet, there is limited research on the shifts of mortality trends and the impact of obesity-related mortality. In this study, we examine mortality trends and the cause-specific proportional composition of deaths by body mass index.

**Methods:**

We used primary healthcare records from the Clinical Practice Research Datalink between 2004 and 2019, linked to national death registration data. There were 880,683 individuals with at least one BMI measurement and a 5-year survival period. We used discrete Poisson regression and joinpoint analysis to estimate the all-cause and cause-specific mortality rate and significance of the trends.

**Findings:**

Between January 1, 2004, and December 31, 2019, all-cause mortality rates declined in the obese category by 3% on average per year (from 23.3 to 14.6 deaths per 1000 person years) in males and 2% on average per year (from 12.5 to 9.4 deaths per 1000 person years) in females. Cardiovascular disease mortality declined 7% on average per year (from 12.4 to 4.4 deaths per 1000 person years) in males and 4% on average per year (from 5.5 to 3.0 deaths per 1000 person years) in females in the obese category. Increases in mortality rates from neurological conditions occurred in all BMI categories in males and females. By the end of the study, cancers became the primary contributor of death in males in all BMI categories and females in the overweight category.

**Interpretation:**

There have been significant declines in all-cause and cardiovascular disease mortality in males and females, leading to a diversification of mortality, with cancers contributing to the highest proportion of deaths and increases in causes such as neurological and respiratory conditions. Further screening, prevention, and treatment implementation for a broader set of diseases is necessary for continued mortality improvements.

**Funding:**

10.13039/501100000761Imperial College London, 10.13039/501100001602Science Foundation Ireland.

**Research in context**

**Evidence before this study**

In high-income countries, all-cause mortality has declined largely due to the declines in cardiovascular disease mortality while the global obesity burden has increased in parallel. Prior research indicates the proportion of deaths from cancers and chronic kidney disease have increased alongside the greater declines in cardiovascular disease mortality in overweight category, whilst no changes were observed for cardiovascular disease mortality in the obese category. The degree to which these changes in causes of mortality have been driven by changes in obesity is unknown. We searched PubMed, without language restrictions, for analyses and reviews from the past 15 years (from January 1, 2008, to December 31, 2023) on the trends of all-cause and cause-specific mortality rates in relation to BMI. We used a combination of the terms for our search: “mortality trends OR changes”, “all-cause mortality”, “cause-specific mortality”, “causes of death”, “BMI”, “body mass index”, “obes∗”, and “overweight”.

**Added value of this study**

To our knowledge, this is the first study to look at the changes in cause-specific mortality rates and corresponding mortality burden across BMI categories for a variety of NCDs. All-cause mortality rates declined the greatest in obese males and females compared to the other BMI categories due to large declines in cardiovascular disease death rates wherein the mortality rate nearly halved between 2004 and 2019 in obese males. As a result, when assessing specific causes of death in males, cancers became the largest contributor to death rates in all BMI categories. Smaller declines for cardiovascular disease mortality occurred in females yet cancers still replaced cardiovascular disease as the largest contributor to death in the overweight category. Similar to prior prevalence studies, non-cancer, non-cardiovascular disease NCDs were the only outcomes with increasing mortality rates, primarily led by increases in neurological and respiratory conditions.

**Implications of all the available evidence**

Cardiovascular disease in males is continuing to decline, resulting in a diversification of causes of death from other NCDs. The decline in cardiovascular disease in females is much smaller, indicating more care needed to reduce risk. However, the changing composition of death indicates the need for more screening and treatment availability for the other NCDs which are increasing proportionally to the total deaths.

## Introduction

Global obesity prevalence among adults has tripled between 1975 (4.7%) and 2016 (13.1%), leading to an estimated 5 million obesity-related deaths in 2019.^1^^,^^2^ The health risks for a number of chronic conditions, such as heart disease and stroke—the leading causes of death worldwide—type 2 diabetes, and certain cancers increase once reaching an overweight range for body mass index (BMI) and continues to increase as BMI levels rise.^3^^,^^4^ For these reasons, the obesity epidemic is thought to have contributed to the persistent burden of non-communicable diseases (NCDs) worldwide and slowed the improvements in morbidity and mortality.

Concurrent with the growth of obesity prevalence, all-cause mortality rates declined by 14% and NCD mortality rates declined by 7% between 2007 and 2017.^5^, ^6^, ^7^ These improvements in mortality rates have largely been driven by declines in cardiovascular disease mortality rates over the past 40 years, resulting in cancer becoming the leading cause of death in some high-income European countries.^8^, ^9^, ^10^, ^11^, ^12^, ^13^, ^14^ However, research suggests the improvements in cardiovascular disease mortality are either slowing down or plateauing.^5^^,^^10^, ^11^, ^12^^,^^15^^,^^16^ For instance, in most high-income countries between 2000 and 2010 cardiovascular disease mortality rates declined by 40%, whereas between 2010 and 2017 rates either increased or declined by less than 2% per year.^12^ Further, the life expectancy improvements which occurred in Western Europe, Japan, and the United States has been slowing or declining since 2011, with the slowdown in England and Wales greater than the other high-income countries.^17^, ^18^, ^19^ The increasing prevalence of overweight and obesity may be a key factor for the stagnation in cardiovascular disease mortality improvements in Australia, Austria, Brazil, Germany, the Netherlands, the UK, and USA.^5^^,^^20^, ^21^, ^22^

Few studies have examined how mortality changes have interacted with or been influenced by the growing prevalence of obesity. Thus, it is unclear whether there has been reductions in mortality and whether these shifts in specific causes of death have been accompanied by decreases or offset by increasing trends in other causes.

This study aims to examine the changes in mortality rates and how the proportional composition of deaths by cause has changed in overweight and obese adults from 2004 to 2019 in England.

## Methods

### Data

This study utilised the Clinical Practice Research Datalink (CPRD) GOLD to examine trends in cause-specific mortality in adults in England across BMI categories. CPRD is a high-quality primary care data source consisting of longitudinal pseudo-anonymised patient data from 986 practices (total number of ever-contributing practices) across the UK, covering nearly three million current patients.^23^ CPRD undertakes comprehensive quality checks of practices and patients, ensuring continuous data collection and follow-up, and regularly submitted data.^24^ The routinely collected data includes demographic, lifestyle, and prescription details; clinical events; preventative care; referrals; and—via linkage to the Hospital Episode Statistics (HES)—hospital admissions and their outcomes.^24^ The patients included in the database are broadly representative of the UK population in terms of age, sex, ethnicity, and BMI distribution based on comparisons to the UK Census and Health Survey for England (HSE).^24^^,^^25^ CPRD was chosen over a dataset such as HSE, as it provides longitudinal BMI measurements for the patients and routinely collected data whereas HSE is cross-sectional.

CPRD provides linkage data-through anonymized patient identifiers-to the Office for National Statistics (ONS), HES, and Index of Multiple Deprivation (IMD).^23^ As ONS only include individuals residing in England, the scope of this study population focuses on English adults the protocol of this study has been approved by the Independent Scientific Advisory Committee and registered with protocol number 21_000310.

### Study cohort

We requested and extracted individuals with at least one BMI measure between 1 January 1999 and 31 December 2014 CPRD. By study design, there are no missing BMI data. In this open cohort, participants with at least one BMI measurement were selected from the study to acquire linkage data from both ONS and HES. These participants then had the following criteria applied: (1) adults aged 18 years or older; (2) a BMI measure between 10 and 70 kg/m^2^; (3) more than one year between GP registration and BMI measure; (4) BMI measurement was not 6 months prior to and 15 months after a pregnancy code; (5) and an acceptable code for the patient and an up to standard code for the practice both as indicated by CPRD. Further detailed inclusion and exclusion criteria are in the Appendix.

To limit reverse causality—the impact of weight loss due to severe illness—we excluded BMI measurements taken before the end of a 5-year follow-up period.^26^ Thus, the final analytical sample consisted of participants who were still being followed up 5 years after a BMI measurement. As an individual was included in the analysis from the time of their first BMI measurement until the end of follow-up, it was possible that one BMI measurement taken on a certain date did not fit the survival requirement, but the prior measurement did. In this scenario, the individual was still included in the analysis, but the BMI which was not within the 5-year survival period was excluded. BMI measurements from 1999 to 2014 were utilised to account for the 5-year survival time. The beginning of the analysis is 1 January 2004 through 31 December 2019. Follow-up began on the date of a participant's first BMI measure and ended at the first date of either death, transferring out of CPRD, or the end of the study. The final study cohort consisted of 880,683 individuals who met the 5-year time requirement subsequent to their BMI measurement.

### Predictors and mortality

BMI was categorised based on WHO classifications as follows: underweight: BMI <18.5 kg/m^2^; normal weight: BMI ≥18.5 and <24.9 kg/m^2^; overweight: BMI ≥25 and <29.9 kg/m^2^; and obese: ≥30 kg/m^2^.^27^ Due to small numbers of participants—and as the analysis is focused on the overweight and obesity groups—the underweight category was included in all models but not shown in the results.

Mortality records for participants fitting the inclusion criteria and with linkage availability were extracted from ONS death registration data. Mortality outcomes were separated into three tiers for a comprehensive list of obesity-related causes of death (Table 1): all-cause mortality (Tier 1), broad categories of mortality (Tier 2), and specific causes of mortality (Tier 3). Tier 2 outcomes consist of cardiovascular diseases; cancers; other NCDs (referred to as non-cancer, non-cardiovascular disease NCDs); and other causes of death. Non-cancer, non-cardiovascular disease NCDs from Tier 2 were broken down into Tier 3 outcomes, which include digestive diseases excluding liver, endocrine, liver, neurological, renal, respiratory, infections, and other causes of death. These were identified by ICD-10 codes from the underlying cause of death listed in the death registration.

**Table 1:** Mortality outcomes of interest by tier and corresponding ICD-10 codes.

Mortality	ICD chapters/codes
**Tier 1 outcomes**	
All-causes	–
**Tier 2 outcomes**	
Non-communicable diseases, excluding cardiovascular disease and cancers	D-H, J-R
Cardiovascular disease	I
Cancer	C
Other	–
**Tier 3 outcomes**	
Respiratory	J, G47.3
Endocrine	E
Neurological	G, F01-03
Liver	K70-77
Renal	N
Digestive excluding liver	K
Infections	A, B, J00-22
Other	–

### Statistical analysis

Discrete Poisson regression models were used to estimate annual all-cause and cause-specific mortality rates by BMI category. Poisson was chosen for the analysis primarily because it allows for the inclusion of more than one time scale, which was necessary as age, BMI, and calendar year are all time-dependent variables. To account for BMI as a time-dependent variable, participants were included in each calendar year using either their first measured BMI from that year or, if BMI was not measured in a respective year, the most recent BMI measurement from a prior year.

A participant's exposure length varied between 0 and 1 year for each year. The exposure length was calculated by dividing the number of days the participant was in the respective calendar year by the total number of days in the year. For example, if a participant died on 12 March 2017, their exposure length for 2017 would be calculated as 71/365. To account for the different exposure lengths of each participant in the study, these values become the offset in the Poisson regression.

Separate models were run for males and females and the covariates included in each model were calendar year, BMI, and age fitted as a three-way interaction term. We then checked the models for over-dispersion, which confirmed Poisson regression was the appropriate model. Age-standardised mortality rates were estimated using 2011 ONS mid-year population estimates as the reference population.^28^ The point estimate (i.e., the mortality rates per 1000 person years) and standard error of each year was input into a joinpoint regression model to calculate the annual average percent change (i.e., the average change in rates per year) and statistical significance of the change. To understand how the cause-specific mortality burden may be changing, proportional mortality—which consists of the proportion each specific cause of death attributed to the total mortality in each BMI category across the study period—was also calculated.

### Sensitivity analyses

Stratified Poisson models were run for smoking status, age, ethnicity, and deprivation levels. Smoking was separated into never smokers and current/ex-smokers. Age was assessed for pre-mature mortality including those 35–75 years of age as mortality in individuals <35 years was small; ethnicity was classified as Asian, Black, Mixed, Other, and White. Deprivation was separated into quintiles with 1 indicating the least deprived and 5 the most deprived. Whilst stratifying for these variables, those who had missing IMD or ethnicity information were excluded from the analysis, thus there was not enough power for the statistical analysis—due to the number of deaths and exposure length per category—so the analysis was only conducted for all-cause mortality.

A sensitivity analysis was run using the ethnicity-specific BMI thresholds, as specified by NICE recommendations. Across Asian and Black ethnicities, BMI categories are as follows: underweight: BMI <18.4 kg/m^2^; normal weight: BMI ≥18.5 kg/m^2^ and <22.9 kg/m^2^; overweight: BMI ≥23 kg/m^2^ and <27.4 kg/m^2^; obese: BMI ≥27.5 kg/m^2^ (63).

A sensitivity analysis using a 2 year exclusion period, rather than the 5 year that was ultimately selected for the main analysis, was also conducted. Finally, the observations from this CPRD dataset were compared to HSE to assess representativeness.

### Role of the funding source

The funder of the study had no role in study design, data collection, data analysis, data interpretation, or writing of the report. MKS and FZ had access to data from CPRD and verified the data reported. MKS had final responsibility for the decision to submit for publication.

## Results

There were 880,683 participants with at least 5 years follow-up after their BMI measurement (Appendix Table S1), contributing to a total of 9,168,223 person-years (Table 2). Females accounted for the majority of patients across all BMI categories, ranging from 76% of underweight to 51% of overweight. Throughout the study, the majority of those who were underweight and normal weight were <44 years of age and female, whilst the majority of those who were overweight or obese were in the 45–64 age group.

**Table 2:** Descriptive statistics, reported as median (IQR) or number (%), throughout a participant's follow-up at each BMI.

	Underweight	Normal weight	Overweight	Obese
	(<18.5 kg/m^2^)	(18.5–24.9 kg/m^2^)	(25–29.9 kg/m^2^)	(>30 kg/m^2^)
	N = 91,622	N = 1,863,420	N = 1,947,244	N = 1,463,039
**Age (Years)**	42 (26, 62)	49 (36, 64)	55 (43, 67)	53 (42, 64)
**Age groups (Years)**				
<44	48,957 (53)	775,769 (42)	546,466 (28)	443,008 (30)
45–64	21,786 (24)	648,588 (35)	829,388 (43)	668,345 (46)
65–74	9488 (10)	241,829 (13)	353,230 (18)	241,519 (17)
75+	11,391 (12)	197,234 (11)	218,160 (11)	110,167 (7.5)
**Gender**				
Male	21,656 (24)	604,970 (32)	945,384 (49)	599,617 (41)
Female	69,966 (76)	1,258,450 (68)	1,001,860 (51)	863,422 (59)
**Ethnicity**				
Asian	3718 (4.1)	46,345 (2.5)	45,411 (2.3)	26,229 (1.8)
Black	836 (0.9)	17,444 (0.9)	23,424 (1.2)	22,717 (1.6)
Mixed	578 (0.6)	8135 (0.4)	6853 (0.4)	5320 (0.4)
Other	1482 (1.6)	25,447 (1.4)	20,280 (1.0)	12,787 (0.9)
White	77,292 (84)	1,602,107 (86)	1,699,308 (87)	1,302,219 (89)
Missing	7716 (8.4)	163,942 (8.8)	151,968 (7.8)	93,767 (6.4)
**Index of multiple deprivation**				
Quintile 1 (Least deprived)	19,563 (21)	511,737 (27)	496,863 (26)	294,950 (20)
Quintile 2	19,424 (21)	437,504 (23)	465,726 (24)	318,096 (22)
Quintile 3	17,469 (19)	354,047 (19)	384,932 (20)	294,931 (20)
Quintile 4	17,896 (20)	313,180 (17)	339,364 (17)	298,797 (20)
Quintile 5 (Most Deprived)	16,703 (18)	233,737 (13)	250,298 (13)	251,086 (17)
Missing	567 (0.6)	13,215 (0.7)	10,061 (0.5)	5179 (0.4)
**Smoking status**				
Current	32,495 (35)	441,181 (24)	356,829 (18)	251,984 (17)
Never	44,152 (48)	1,034,823 (56)	1,044,518 (54)	771,256 (53)
Ex	10,652 (12)	346,703 (19)	506,897 (26)	404,278 (28)
Missing	4323 (4.7)	40,713 (2.2)	39,000 (2.0)	35,521 (2.4)
**Drinking status**				
Current	55,660 (61)	1,410,725 (76)	1,516,331 (78)	1,060,295 (72)
Never	17,868 (20)	246,314 (13)	248,459 (13)	237,279 (16)
Ex	1849 (2.0)	25,904 (1.4)	31,142 (1.6)	30,231 (2.1)
Missing	16,245 (18)	180,477 (9.7)	151,312 (7.8)	135,234 (9.2)
**Follow-up**				
Years	9.0 (6.9, 12.1)	9.8 (7.2, 13.0)	9.9 (7.4, 13.1)	9.9 (7.3, 13.1)
Deaths	9.8%	6.3%	7.1%	6.7%
Age at death	82 (74, 89)	83 (75, 89)	81 (74, 87)	78 (70, 84)

Unless otherwise stated, estimates are reported as median (IQR) or number (%).

Across all BMI categories, the majority of individuals were of white ethnicity. Over 25% of individuals in the normal weight and overweight categories and 20% of individuals in the obese category were in the least deprived IMD quintile. In comparison, 13% of individuals in the normal weight and overweight categories and 17% of individuals in the obese category were in the least deprived category. The proportion of current smokers was lower in the overweight (18%) and obese (17%) categories compared to the normal weight (24%) category, whereas ex-smokers were higher in the overweight (26%) and obese (28%) categories compared to the normal weight (19%) category. Small differences were observed for all drinking categories across the BMI categories (excluding underweight).

Similarities between the crude prevalence of overweight, and overweight and obesity by IMD, when comparing the CPRD dataset used in this analysis and HSE, indicate representativeness of the dataset (Appendix Figure 2).^29^^,^^30^ Further, throughout the study period, the demographics of participants remained fairly consistent, though the proportion of males increased as the study period progressed, in addition to increasing obesity prevalence (Appendix Table 2).

### All-cause mortality

**Fig. 1:**
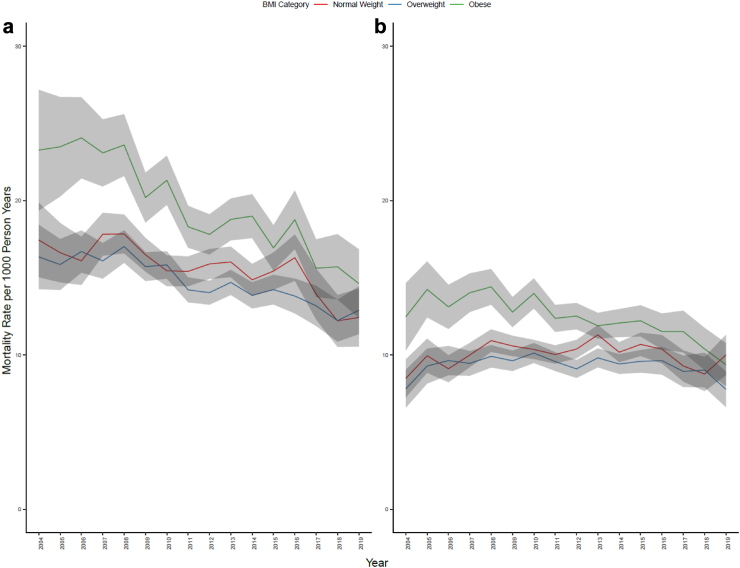
**Age-adjusted all-cause mortality rates in (a) males and (b) females**.

From 1 January 2004 to 31 December 2019, males and females with obesity had higher rates of all-cause mortality than overweight or normal weight individuals but also experienced the greatest mortality decline over time (Table 3; Fig. 1). In males with obesity, death rates declined from 23 to 15 deaths per 1000 person years, resulting in an average decline of 3% per year (95% CI: −4.2%, −2.2%) between 2004 and 2019. Death rates declined by about 2% per year for both normal weight (95% CI: −4.4%, −0.1%) and overweight (95% CI: −2.8%, −1.3%) categories (Table 3).

**Table 3:** Age-adjusted all-cause mortality (tier 1), tier 2, and tier 3 average percent change in death rate across BMIs between 2004 and 2019, by sex.

	Normal weight	Overweight	Obese
	Average change per year (%)	Average change per year (%)	Average change per year (%)
**Males**			
**Tier 1**			
All-cause	−2.5 (−4.4, −0.1)	−2 (−2.8, −1.3)	−3.2 (−4.2, −2.2)
**Tier 2**			
Non-cancer, non-cardiovascular disease NCDs	0.5 (−1.5, 2.6)	1.8 (0.2, 3.5)	0.5 (−1.2, 2.3)
Cardiovascular disease	−4.6 (−5.4, −3.8)	−5.1 (−6.1, −4.1)	−6.8 (−8.4, −5.4)
Cancer	−1.2 (−3.2, 0.7)	−1 (−4.5, 1.8)	−1.8 (−3.6, 0.1)
Other	−0.3 (−5.1, 4.5)	−1.3 (−3.4, 0.9)	−1.7 (−4.6, 1.3)
**Tier 3**			
Cancers	−1.1 (−3.2, 0.8)	−1 (−4.4, 1.5)	−1.7 (−3.5, 0.1)
Cardiovascular disease	−4.6 (−5.4, −3.8)	−5.1 (−6.1, −4.1)	−6.7 (−8.2, −5.2)
Digestive excluding liver	−1 (−5.3, 3.3)	−2.4 (−6.3, 1.7)	−0.9 (−8.1, 7.1)
Endocrine	−11.8 (−18.1, −5.2)	−7.7 (−17.2, 2.7)	−4.4 (−10, 1.5)
Infections	−1.7 (−4.8, 1.3)	0.4 (−1.9, 2.8)	1.4 (−2.7, 5.7)
Liver	−89.6 (−95.7, −83.5)	−4 (−9.4, 1.5)	8.1 (−13.1, 25.2)
Neurological	9.1 (3.4, 13.7)	7.2 (3, 11.8)	304.7 (122.7, 550.2)
Other	5.6 (−2, 11.3)	2.2 (−1.9, 6.3)	−3.8 (−11.8, 5.4)
Renal	0.6 (−13.9, 11.5)	−3.3 (−10.4, 4.2)	−1.3 (−8.6, 6.9)
Respiratory	−0.3 (−2.6, 1.9)	1.2 (−1.2, 3.6)	−0.5 (−3.3, 2.5)
**Females**			
**Tier 1**			
All-cause	−0.2 (−2.9, 3.1)	0 (−1.3, 1.2)	−2.1 (−3, −1.2)
**Tier 2**			
Non-cancer, non-cardiovascular disease NCDs	3.6 (1.6, 5.9)	6.2 (4.3, 8.2)	0.1 (−1.7, 1.9)
Cardiovascular disease	−2.4 (−4.3, −0.6)	−3.6 (−4.9, −2.5)	−4.4 (−6.2, −2.7)
Cancers	0 (−1.6, 1.2)	0.1 (−3, 2.9)	−1.1 (−4.7, 2.7)
Other	−0.8 (−3.4, 1.9)	−2.1 (−5.6, 1.2)	−1.6 (−5.9, 2.7)
**Tier 3**			
Cancers	−0.9 (−2.1, 0.3)	0.1 (−3, 3.1)	−1.1 (−4.6, 2.5)
Cardiovascular disease	−2.6 (−4.5, −0.6)	−3.7 (−5, −2.5)	−4.4 (−6.2, −2.6)
Digestive excluding liver	−1.6 (−4.5, 1.5)	−1.6 (−6.9, 4.1)	−1 (−4.3, 2.5)
Endocrine	−33.4 (−73.2, 63.4)	−2.5 (−7.8, 3.1)	−11.1 (−15.7, −6.3)
Infections	0.8 (−1.9, 3.5)	9.5 (3, 16.6)	−0.2 (−3.2, 2.9)
Liver	−30.5 (−71.9, 68.7)	−3.1 (−10.7, 5.3)	976 (509.3, 2408.4)
Neurological	17.4 (14.9, 19.9)	15.4 (10.4, 20.5)	5 (−0.5, 12.9)
Other	3.8 (0.8, 7)	−0.4 (−5, 4.4)	−0.5 (−5.4, 4.6)
Renal	−0.2 (−7, 6.9)	0.4 (−14.2, 16.3)	−3.2 (−13.5, 8.3)
Respiratory	−0.4 (−6, 5.3)	1.2 (−2.1, 4.6)	5.3 (−1.1, 9.6)

NCD, Non-communicable disease.

The death rates for females declined in the obese category from 13 to 9 deaths per 1000 person years, an average decline of 2% per year (95% CI: −3.0%, −1.2%), but did not change in the normal weight (8–10 deaths per 1000 person years) or overweight (8–8 deaths per 1000 person years) categories (Table 3).

### Deaths due to cardiovascular disease, cancer, and all other NCDs combined

**Fig. 2:**
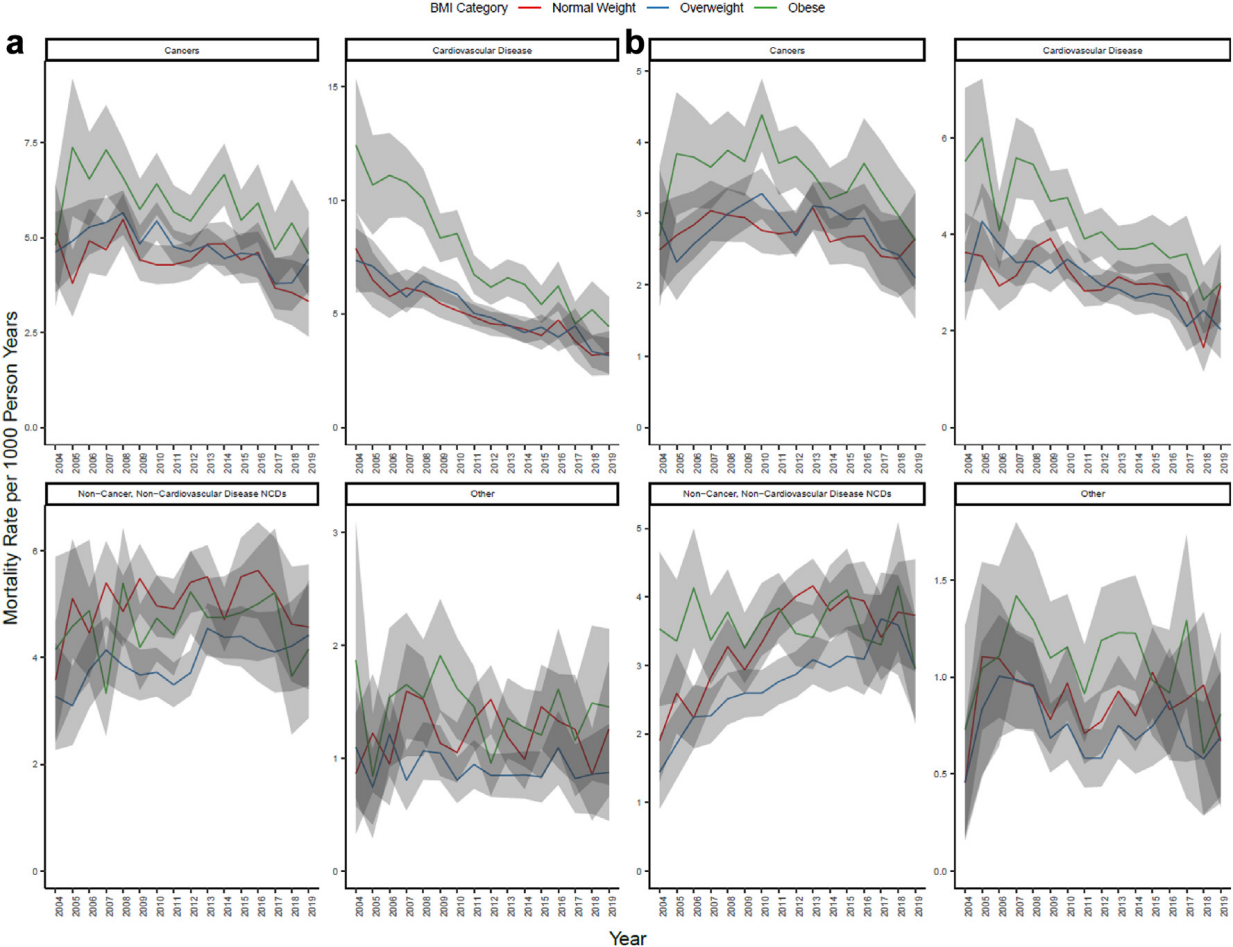
**Age-adjusted mortality rates in Tier 2 outcomes in (a) males and (b) females**.

Among the Tier 2 causes of death in males, the death rates from cardiovascular disease significantly declined in a similar magnitude across all BMI categories (Fig. 2; Table 3). In males, cardiovascular disease mortality rates steadily declined in the obese category by 7% per year (95% CI: −8.4%, −5.4%) and 5% per year in the overweight (95% CI: −6.1, −4.1%) and normal weight (95% CI: −5.4%, −3.8%) categories. For non-cancer, non-cardiovascular disease NCD mortality in males, normal weight and overweight categories had similar death rates and magnitude of change in 2004 and 2019. However, the mortality rate in the overweight category increased by 2% per year (95% CI: 0.2%, 3.5%) and no change occurred in the normal weight or obese category. No changes occurred throughout the study period for cancer mortality.

In females, cardiovascular disease mortality rates declined in all groups, with a 4% decline per year in the obese (95% CI: −6.2%, −2.7%) category and a 3% decline per year in the overweight (95% CI: −4.9%, −2.5%) and normal weight (95% CI: −4.3%, −0.60%) categories (Fig. 2b). There was no change in cancer mortality in any of the BMI categories (Fig. 2b; Table 3). Across non-cancer, non-cardiovascular disease NCD mortality, a 6% increase per year (95% CI: 4.3%, 8.2%) occurred in the overweight category and a 4% increase per year (95% CI: 1.6%, 5.9%) in the normal weight category. There were no changes in non-cancer, non-cardiovascular disease NCDs mortality rates in the obese category.

### Specific causes of mortality

In males, increases in neurological deaths were observed for all BMI categories. In the obese category, mortality rates increased from <0.0001 to 0.87 per 1000 person years, accounting for a 305% increase per year (95% CI: 122.7%, 550.2%). The increases in death rates per year were approximately 7% in the overweight weight (95% CI: 3.0%, 11.8%) and 9% in the normal weight (95% CI: 3.4%, 13.7%) categories (Table 3; Appendix Figure 3A). Death rates declined in the normal weight category for liver diseases by 90% per year (95% CI: −95.7%, −83.5%) and for endocrine conditions by a 12% per year (95% CI: −18.1%, −5.2%), with no changes in the overweight or obese categories for either causes of death. No changes were observed for digestive disease excluding liver diseases, renal conditions, or respiratory disease in any of the BMI categories.

In females, deaths due to neurological conditions increased in the normal weight and overweight BMI categories, with no changes in the obese category (Table 3, Appendix Figure 3B). Neurological mortality rates increased by 17% per year (95% CI: 14.9%, 19.9%) in the normal weight category and 15% per year (95% CI: 10.4%, 20.5%) in the overweight category. For liver disease in females in the obese category, the mortality rates increased from <0.0001 to 0.042 per 1000 person years, accounting for a 976% increase per year (95% CI: 509.3%, 2408.4%), whereas there were no changes in the normal weight or overweight categories. In the obese category, mortality rates for endocrine disease declined by 11% per year (95% CI: −15.7%, −6.3%). Otherwise, no changes were observed in females for non-cancer, non-cardiovascular disease NCD mortality rates.

### Proportional mortality

**Fig. 3:**
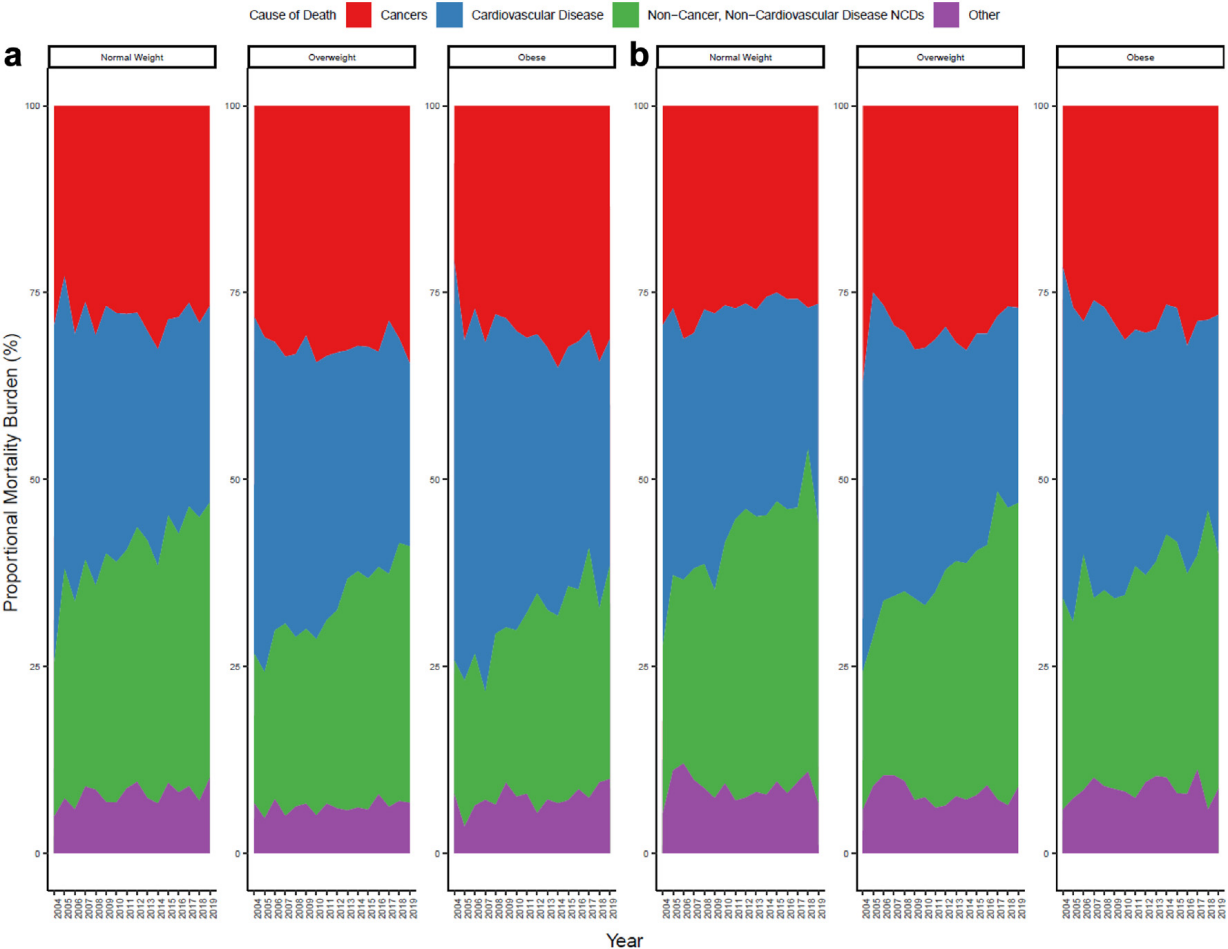
**Proportional composition of Tier 2 outcomes between 2004 and 2019 in (a) males and (b) females**.

Across the BMI groups in males, the proportion of all deaths that were due to cardiovascular disease declined, ranging from 45% to 54% of deaths across groups in 2004 to 25%–30% across groups in 2019 (Fig. 3a; Appendix Table 3). Between 2004 and 2019, the contribution of deaths from cancers declined from 30% to 27% in the normal weight category but increased from 28% to 35% in the overweight category and from 21% to 31% in the obese category. The proportion of deaths from non-cancer, non-cardiovascular disease NCDs increased in all BMI categories: from 21% to 37% in the normal weight category; from 20% to 34% in the overweight category; and from 18% to 29% in the obese category.

In females, the proportion of deaths due to cardiovascular disease declined from 39% in 2004 to 26% in 2019 in the overweight category (Fig. 3b). Cardiovascular disease mortality declined, accounting for 43%–44% of deaths in 2004 for both the normal weight and obese category to 30% in the normal weight category and to 32% in the obese category by 2019 (Appendix Table 3). Between 2004 and 2019, the proportions of deaths from cancer differed across BMI categories. Decreases occurred in the overweight category from 37% to 27%, declined in the normal weight category from 30% to 27% and increased in the obese category from 22% to 28%. The proportion of deaths due to non-cancer, non-cardiovascular disease NCDs increased in all BMI categories, but the greatest increase was in the overweight category from 19% in 2004 to 38% in 2019.

The changes in the mortality contributions for cause-specific mortality across males, and how they ranked between 2004 and 2019, are shown in Appendix Table 4. In males, large increases in contributions of mortality were observed for neurological conditions from nearly 0% to 6% in the obese category, from 2% to 9% in the normal weight category, and from 4% to 11% in the overweight category (Appendix Figure 4A). Other increases occurred for respiratory mortality from 7% to 14% in the normal weight category, from 7% to 11% in the overweight category, and from 4% to 7% in the obese category (Appendix Table 4). The proportion of deaths from digestive diseases excluding liver increased from 3% to 6% in both normal weight and obese categories but decreased in the overweight category.

In females, changes in contributions of cause-specific mortality have changed in rankings as shown in Appendix Table 5. Increases in the proportion of deaths due to neurological conditions occurred in overweight from 2% to 16%, normal weight from 1% to 13%, and obese from 4% to 9% (Appendix Figure 4B). The proportion of deaths due to respiratory disease in the obese category increased from 3% to 7%, in the overweight category increased from 7% to 9% and in the normal weight category increased from 6% to 7% (Appendix Table 3).

### Sensitivity analyses

When the assessing mortality rate trends by smoking status (current/ex-smokers and never smokers) the trend lines were similar for both groups, though the mortality rate was higher amongst current/ex-smokers and rate of decline was greater in never smokers (Appendix Figures 5–10). An analysis only accounting for pre-mature mortality (aged 35–74 years) was also conducted and shown in Appendix Figures 11–13. The sensitivity analysis using a 2 year exclusion period to estimate mortality rates by all-cause and cause-specific outcomes is in Appendix Figures 14–16. These analyses show a much higher and less likely mortality rate per 1000 person years in normal weight compared to overweight and obese categories, justifying the use of a five year exclusion period. Ethnicity-specific BMI categories were implemented as a supplementary analysis for all-cause mortality and no distinct difference was observed for mortality trends. In this study, we were able to explore the relationship between obesity and mortality rates by IMD and ethnicity but only for all-cause mortality due to the smaller sample size after stratification occurred.

## Discussion

In this English population-based study investigating the changing mortality spectrum associated with overweight and obesity, we highlight three main findings. Firstly, all-cause mortality is declining, with the greater declines in obese males and females than in those who were overweight or normal weight. For males, this reduced the all-cause mortality rate by 37% and cardiovascular disease mortality rate by 64%, as declines were driven by significant reductions in cardiovascular disease mortality. Secondly, in Tier 2 outcomes, non-cancer, non-cardiovascular disease NCDs are the only outcomes with increasing mortality rates. Finally, cancers have become the primary contributor of death in males in all BMI categories, and among females in the overweight category.

Our findings are consistent with previous research indicating that there have been steady declines in both all-cause mortality and cardiovascular disease mortality.^8^^,^^11^^,^^31^ Some research suggests that increasing obesity prevalence is either slowing or offsetting the reduction in cardiovascular disease rates, but we found no stagnation in mortality rates in this large sample of the English populations.^8^^,^^12^^,^^13^ Thus, despite the continued growth of obesity, improvements in treatment, risk factor management, and/or lifestyle changes appear to have offset or negated the impact of obesity as a risk factor for cardiovascular disease.^31^

Mortality rates for cardiovascular disease and cancer narrowed over the study period for all BMI categories. Although there has been a 19% decline in cancer mortality rates in the UK between 1971 and 2019, changes in cancer rates within BMI categories were not significant.^32^^,^^33^ Throughout the study period, the proportion of deaths from cardiovascular disease declined in all BMI categories and was replaced by deaths from cancers and non-cancer, non-cardiovascular disease NCDs. The large declines in cardiovascular disease mortality were sufficient for cancer to replace cardiovascular disease as the leading cause of death in Tier 3 outcomes in most subgroups.^15^^,^^32^^,^^33^ Cancer overtook cardiovascular disease as the leading cause of death in the overweight females in Tier 3 outcomes.

The increase in neurological mortality is also a primary contributor to this increase, as expected, as literature indicates the number of deaths from neurological conditions increased 39% in England since 2001.^34^ In this analysis, the proportion of deaths from neurological conditions, primarily consisting of dementia and Alzheimer's disease, is one of the main reasons for the diversification of mortality from cardiovascular disease to non-cancer, non-cardiovascular disease NCDs. This is consistent with previous trend studies, with the proportion of deaths from dementia and Alzheimer's deaths in the UK increasing from 5% in 2001 to 13% in 2018.^31^ However, the mortality risk from dementia and Alzheimer's and its association to overweight and obesity is unclear. Obesity in mid-life is an established risk factor for dementia but obesity at older ages shows an inverse association with dementia risk.^35^ Further studies have shown that the seemingly protective effect of obesity on dementia risk disappears when using a BMI from more than 10 years prior to a diagnosis, due to reverse causation from weight loss in the pre-clinical stages of dementia.^36^^,^^37^ In our findings the death rates were highest in both overweight males and females and proportion of deaths from neurological conditions increased the most in the obesity and overweight categories.

A strength of this study is the use of CPRD, a large, dataset that is generally representative of the English population. With the longitudinal, real-world data provided by CPRD we were able to use BMI as a time-dependent variable and account for any changes throughout the study period, allowing us to analyse the trends in mortality through discrete Poisson regression models, which would not have been an option if we used HSE as the data collected is cross-sectional. However, if a participant was still being followed-up but did not have a BMI measure recorded that year, we had to assume the latest BMI measured prior to that calendar year had not changed.

Data is routinely collected from the GP practices and linked to ONS to provide comprehensive real-world data. This also comes with limitations. People who go to the GP regularly are more likely to have health conditions and females aged 16–60 years old are more likely to seek health services than males.^38^ Whilst there were about 20 million patients included in CPRD at the time of extraction, nearly 6.1 million were extracted having at least one BMI measurement in our study period, indicating a potential selection bias.^23^ The proportion of individuals with a BMI measurement within CPRD has improved, with 37% of individuals having at least one BMI measurement in 1990–1994, increasing to 77% by 2005–2011.^24^ Yet, the proportion of those with a BMI measurement increases with increasing age, up to 75 years old, and was greater in individuals with type 2 diabetes, schizophrenia/psychoses, and statin users.^24^ Our dataset was restricted to persons with a BMI measurement, so we could not look at whether there are considerable differences between patients who get their BMI measured compared to those who do not.

In an ancillary evaluation of the representativeness of our CPRD sample, we found that the prevalence of overweight and obesity in CPRD is similar to that of HSE, which is representative of the population in England. However, when stratifying by IMD, we found in CPRD there is a slight under-representation of males in the overweight category in the more deprived IMD quintiles and an over-representation of overweight and obese males in the least deprived IMD quintiles. These differences were not observed in females, who had very similar prevalence of overweight and obesity by IMD when comparing the two datasets. Research does suggest that females and those who are more deprived are more likely to visit the GP, whereas those who are more deprived are more likely to have anthropometric measurements.^39^^,^^40^

BMI is a multifaceted condition often criticised for not directly assessing body fat or differentiating between lean or fat mass. Measuring techniques such as bioelectrical impedance and dual-energy X-ray absorptiometry are considered the gold standards as they directly measure body mass, but they are impractical for routine use.^41^ Further, using measures for central obesity such as waist circumference, are as highly correlated to BMI and show similar risk of disease, thus The National Institute for Health and Care Excellence guidelines specify waist circumference should be a secondary measurement to BMI.^42^^,^^43^ Thus, BMI has become the standard, being an appropriate and reliable measurement to estimate the risk of morbidity and mortality associated to obesity.

Another limitation is that policy changes have been implemented during this study. The Quality and Outcomes Framework was introduced in 2004 in UK primary care where GP practices were financially incentivised for the attainment of specific quality indicators.^44^ However, when comparing the descriptive statistics by year, no stark differences were observed for the variables we considered in the study from 2004 through 2019. Secondly, how the primary cause of death is defined could contribute to the increase in mortality rates from certain causes of death, and their proportional contribution. For instance, from 2014 an increased policy focus on dementia diagnosis and care resulted in higher recording of dementia as the underlying cause of death more often than previous years.^45^ Similar trends are occurring in the United States and Australia wherein dementia is being reported as the underlying cause of death rather than the secondary cause, and has resulted in large increases in dementia mortality rates, and a concurrent decline in cardiovascular disease mortality.^46^ We are unable to account for changes in policy and coding practices in the analysis but recognizing this is as a potential reason for the increase in neurological mortality rates, for instance, is important in the interpretation of the results.

Reverse causality is a concern in any study of BMI and mortality. There is clear evidence that body composition changes with age.^47^ In individuals 65 and older, all-cause mortality risk is reduced with a BMI between 23 kg/m^2^ and 29 kg/m^2^, but is increased with a BMI under 22 kg/m^2^.^48^ As illness and aging can impact weight loss, and to ensure these factors were not causing participants to lose weight prior to death, we selected a 5-year survival period over 2-year to ensure reverse causality was accounted for. However, there is the possibility that participants had lost weight more than five years prior to their death due to illness, or from aging. Further, the 5-year exclusion period significantly reduced the sample size and is a likely contributor to low death rates; a larger sample size to allow more precise death rates, whilst still accounting for reverse causality, would be ideal.

Despite these limitations, this analysis provides useful insights into the pattern of changing overweight and obesity-related mortality and provided insight on future work which would be impactful. Considering the large declines in cardiovascular disease mortality, investigating pre-mature cardiometabolic mortality utilising each condition reported on the death certificate would greatly contribute to the growing amount of research on cardiovascular disease mortality trends and obesity.

Stagnation in all-cause and cardiovascular disease mortality observed in prior studies from 2011 onwards is largely due to the increasing obesity prevalence, but also from economic recession and austerity policies.^8^^,^^12^^,^^13^^,^^49^^,^^50^ Public health system cuts were greater in the poorer areas of England, resulting in a serious health crisis with health inequalities widening.^49^ Individuals in the most deprived areas of England are more likely to have a shorter life expectancy and spend more of their life with ill health, compared to the least deprived areas.^49^ Further, life expectancy declined between 2010–12 and 2016–2018 in the most deprived areas of England in females, in contrast to an increase in the least deprived areas.^51^ In males, whilst an life expectancy increased for all IMD categories–with a negligible increase in the most deprived areas–the magnitude of increase was the lowest in the most deprived compare to the least deprived.^51^ We did not have the statistical power in this study to investigate the intricacies of IMD, BMI, and mortality trends, but it would be a major contribution to the existing research.

In summary, between 2004 and 2019 there were large declines in all-cause and cardiovascular disease mortality in males and females in England, with the greatest reductions among obese individuals driven by the reduction in deaths from cardiovascular disease. Non-cancer, non-cardiovascular disease mortality rates increased for normal weight and overweight categories, but not the obese category in males or females. Once specific NCDs were analysed, cancers became the leading contributor of deaths amongst males in all BMI categories and females in the overweight category, whilst the proportion of deaths from neurological, respiratory, and digestive excluding liver disease also increased. The causes of death becoming more diverse among overweight and obese individuals in the general population indicate that clinical and preventative strategies should be adjusted to account for a broader set of diseases.

## Contributors

MKS and EWG had the initial idea for the paper. MKS led the analysis with input from FZ, YJC, EPV, NH. MKS wrote the first draft of the manuscript with input from all co-authors.

## Data sharing statement

Data from this study will not be made available as CPRD requires an application and permissions to access patient level data.

## Declaration of interests

FZ reports a consultancy fee from Daiichi Sankyo; NH is funded by NHS England.
