# Supplementary material for: Trends in all-cause and cause-specific mortality by BMI levels in England, 2004–2019: a population-based primary care records study
Source: Lancet Reg Health Eur. 2024 Jul 2;44:100986. doi: 10.1016/j.lanepe.2024.100986 (PMC11268361; doi:10.1016/j.lanepe.2024.100986)
Supplement: Supplementary Material [file mmc1.pdf]

Supplementary appendix

Table of contents

	Page
**Additional methods**	2
**Appendix Figure 1.** Comparing the final analytic sample of this study from CPRD to Health Survey for England: a) The crude prevalence of overweight or obesity and b) The crude prevalence of overweight and obesity by Index of Multiple Deprivation[29,30]	3
**Appendix Figure 2.** Exclusion criteria applied at Phase 1 (only applied to the CPRD Gold dataset) and Phase 2 (after randomly selecting a subset of participants and linking the CPRD Gold dataset to ONS death registration data) of data cleaning	4
**Appendix Table 1.** Reason and proportion of participants not included in the 5-year post-BMI period	5
**Appendix Table 2.** Descriptive statistics of all participants, every 5 years throughout the follow-up period	6
**Appendix Figure 3.** Age-adjusted mortality rates in Tier 3 outcomes between 2004 and 2019 in (a) males and (b) females	7
**Appendix Table 3.** Changes in proportional mortality (percent) for Tier 3 outcomes in 2004 and 2019 per 1000 person years and cumulative change in death rate across BMIs, by sex	8
**Appendix Figure 4.** Proportional composition of Tier 3 outcomes between 2004 and 2019 in (a) males and (b) females	9
**Appendix Table 4.** Tier 3 outcomes and the proportional contribution (%) of mortality rankings in 2004 and 2019 by BMI in males	10
**Appendix Table 5.** Tier 3 outcomes and the proportional contribution (%) of mortality rankings in 2004 and 2019 by BMI in females	11
**Appendix Figure 5.** Age-adjusted all-cause mortality rates in never smokers between 2004 and 2019 in (a) males and (b) females	12
**Appendix Figure 6.** Age-adjusted all-cause mortality rates in current/ex-smokers between 2004 and 2019 in (a) males and (b) females	13
**Appendix Figure 7.** Age-adjusted mortality rates in never smokers across Tier 2 outcomes between 2004 and 2019 in (a) males and (b) females	14
**Appendix Figure 8.** Age-adjusted mortality rates in current/ex-smokers across Tier 2 outcomes between 2004 and 2019 in (a) males and (b) females	15
**Appendix Figure 9.** Age-adjusted mortality rates in never smokers across Tier 3 outcomes between 2004 and 2019 in (a) males and (b) females	16
**Appendix Figure 10.** Age-adjusted mortality rates in current/ex-smokers across Tier 3 outcomes between 2004 and 2019 in (a) males and (b) females	17
**Appendix Figure 11.** Age-adjusted all-cause mortality rates in 35-74 year olds between 2004 and 2019 in (a) males and (b) females	18
**Appendix Figure 12.** Age-adjusted mortality rates in 35-74 year olds across Tier 2 outcomes between 2004 and 2019 in (a) males and (b) females	19
**Appendix Figure 13.** Age-adjusted mortality rates in 35-74 year olds across Tier 3 outcomes between 2004 and 2019 in (a) males and (b) females	20
**Appendix Figure 14.** Sensitivity analysis with a two-year exclusion period: age-adjusted mortality rates in Tier 1 outcomes in (a) males and (b) females	21
**Appendix Figure 15.** Sensitivity analysis with a two-year exclusion period: age-adjusted mortality rates in Tier 2 outcomes in (a) males and (b) females	22
**Appendix Figure 16.** Sensitivity analysis with a two-year exclusion period: age-adjusted mortality rates in Tier 3 outcomes in (a) males and (b) females	23

Additional methods

Initial data cleaning of CPRD dataset

Patients were excluded for a few administrative reasons. If a patient's first registration date was not the same as the current registration date they were excluded, as this may indicate gaps in the registration and temporary patients. The purpose of this was to only include permanent patients at the practice to acquire the most longitudinal data available and most accurate BMI measurement data throughout follow up. Patients who had a BMI measurement within 12 months of their registration date at the practice were also excluded. Newly registered patients at the general practice often get their BMI measured for administrative purposes, but for this research we only wanted BMI measures that were taken for medical purposes at the clinician's discretion.

The plausibility range for BMI was 10-70 kg/m^2, excluding any extreme outliers for both abnormally low and high BMIs. Further, only adults aged 18 years and older were included, but this was an open cohort study so once a patient turned 18 years old and fit the other criteria, they were then included in the study.

As preparing for pregnancy and being pre- and post-natal influences lifestyle and behaviours, in addition to weight gain throughout pregnancy, cleaning for pregnancy in the dataset was imperative. Using a website called CALIBER, now replaced by Health Data Research UK, I searched read codes which indicated pregnancy and extracted them. I then matched the patients in my dataset that had a pregnancy read code, and using the date a pregnancy read code was entered and the date of a BMI measurement, I excluded BMI measurements which were taken 6 months prior to pregnancy and 15 months after pregnancy.

There were also certain cases where the gender was coded as neither male nor female, so they were excluded.

Preparing for linked data

Prior to requesting linked data (ONS, HES, and IMD), the start and end of follow up for each patient was established using the CPRD dataset to ensure the BMI measurements were within the defined study period (1 January 1999 – 31 December 2019) and other dates of interest. The start of follow up was the latest date of the following: practice up to standard date, registration date, linkage availability date, or the study start date (1 January 1999). The end of follow up was the earliest date of the following: transferring out of practice date, date of death, last collection date of the practice, end of linked data availability, or the end of the study period (31 December 2019). Whilst data was acquired until November 2021, due to the COVID-19 epidemic, the end of follow up was December 2019 to ensure mortality rates and causes of death were not skewed.

Linked data had to be requested for the subset of patients that had it available to them. For analytical simplicity, 1,500,000 random patients were selected to get linkage data. To choose these participants, two new variables were created consisting of random normal distribution values. Once the patient IDs were sorted in ascending order, the first 1,500,000 participants were selected. The linkage data for these participants were requested and received.

Despite the CPRD dataset containing date of death for patients, the ONS death data is the most reliable source for this information, thus the end of follow up for participants was updated once receiving linkage data. The same criteria were applied, but instead using the ONS date of death that was provided rather than CPRD-provided date of death.

Once these inclusion and exclusion criteria were applied and participants were identified based on the start and end of follow up, there were 1,495,193 participants with linkage data included in the study population. The simplified flowchart of inclusion/exclusion criteria are shown in Appendix Figure 2.

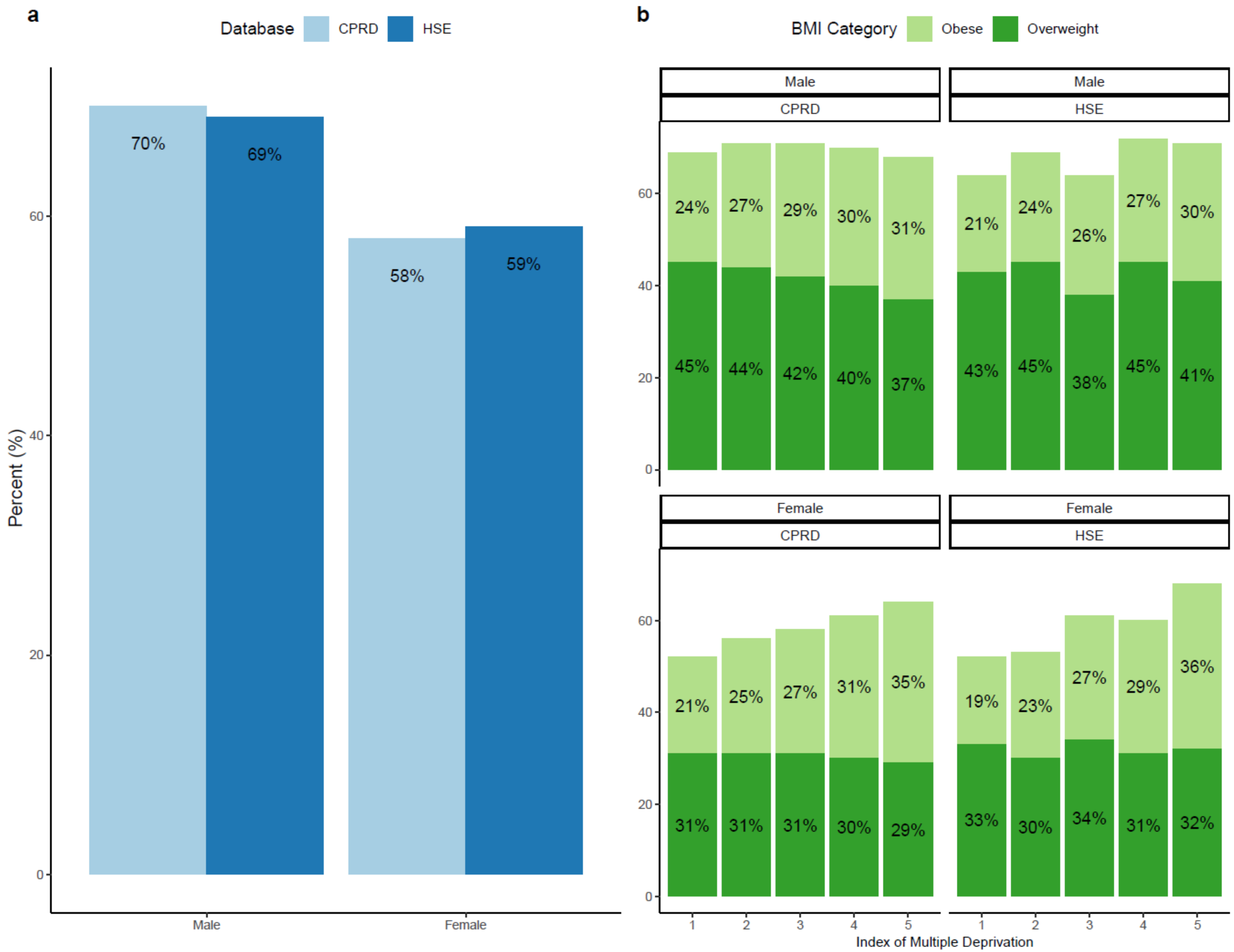

Appendix Figure 1. Comparing the final analytic sample of this study from CPRD to Health Survey for England: a) The crude prevalence of overweight or obesity and b) The crude prevalence of overweight and obesity by Index of Multiple Deprivation[29,30]

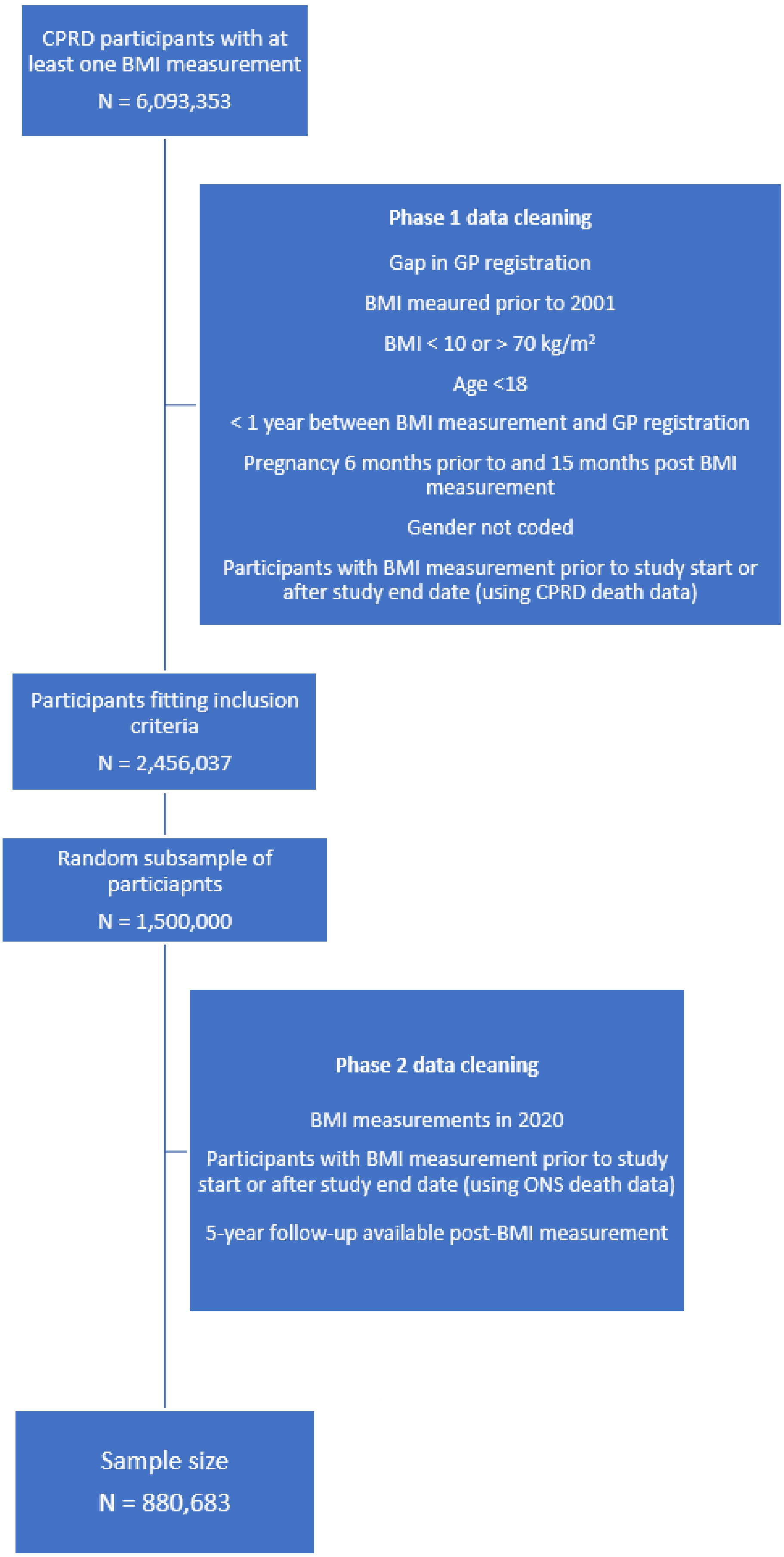

Appendix Figure 2. Exclusion criteria applied at Phase 1 (only applied to the CPRD Gold dataset) and Phase 2 (after randomly selecting a subset of participants and linking the CPRD Gold dataset to ONS death registration data) of data cleaning

Appendix Table 1. Reason and proportion of participants not included in the 5-year post-BMI period

	Underweight	Normal Weight	Overweight	Obese
Death	6,395 (1·3%)	42,801 (8·4%)	37,872 (7·4%)	24,755 (4·8%)
End of study period	202 (0·04%)	2,464 (0·48%)	2,351 (0·46%)	1,798 (0·35%)
Transferred out	12,583 (2·5%)	158,657 (31·1%)	126,638 (24·8%)	94,030 (18·4%)

Appendix Table 2. Descriptive statistics of all participants, every 5 years throughout the follow-up period

	2004<br>N = 103,258	2009<br>N = 427,695	2014<br>N = 489,669	2019<br>N = 151,409
**Age (Years)**				
<44	33%	33%	34%	33%
45-64	41%	40%	40%	42%
65-74	16%	16%	15%	16%
75+	10%	10%	10%	8·80%
Median (IQR)	53 (40, 65)	53 (40, 65)	52 (40, 65)	52 (41, 65)
**BMI (kg/m^2)**				
Underweight	1·80%	1·70%	1·70%	1·60%
Normal Weight	37%	35%	34%	33%
Overweight	36%	36%	36%	36%
Obese	25%	26%	28%	29%
Median (IQR)	26·2 (23·3, 29·9)	26·5 (23·5, 30·2)	26·7 (23·6, 30·4)	26·8 (23·7, 30·8)
**Gender**				
Male	35%	40%	42%	43%
Female	65%	60%	58%	57%
**Ethnicity**				
Asian	1·60%	1·90%	2·60%	2·90%
Black	0·80%	0·90%	1·40%	1·90%
Mixed	0·20%	0·30%	0·50%	0·60%
Other	0·70%	0·90%	1·30%	1·60%
White	89%	89%	86%	84%
Missing	7·80%	7·40%	7·90%	9·40%
**Index of Multiple Deprivation**				
Quintile 1 (Least Deprived)	23%	24%	24%	27%
Quintile 2	24%	24%	23%	22%
Quintile 3	21%	20%	20%	17%
Quintile 4	18%	18%	18%	17%
Quintile 5 (Most Deprived)	15%	14%	14%	14%
Missing	0·20%	0·40%	0·50%	1·90%
**Smoking status**				
Current	22%	21%	19%	18%
Never	57%	53%	54%	56%
Ex	18%	23%	25%	25%
Missing	3·80%	3·20%	1·20%	0·50%
**Drinking status**				
Current	77%	77%	74%	74%
Never	15%	13%	14%	15%
Ex	0·70%	1·40%	2·00%	2·30%
Missing	7·60%	8·10%	9·80%	8·50%

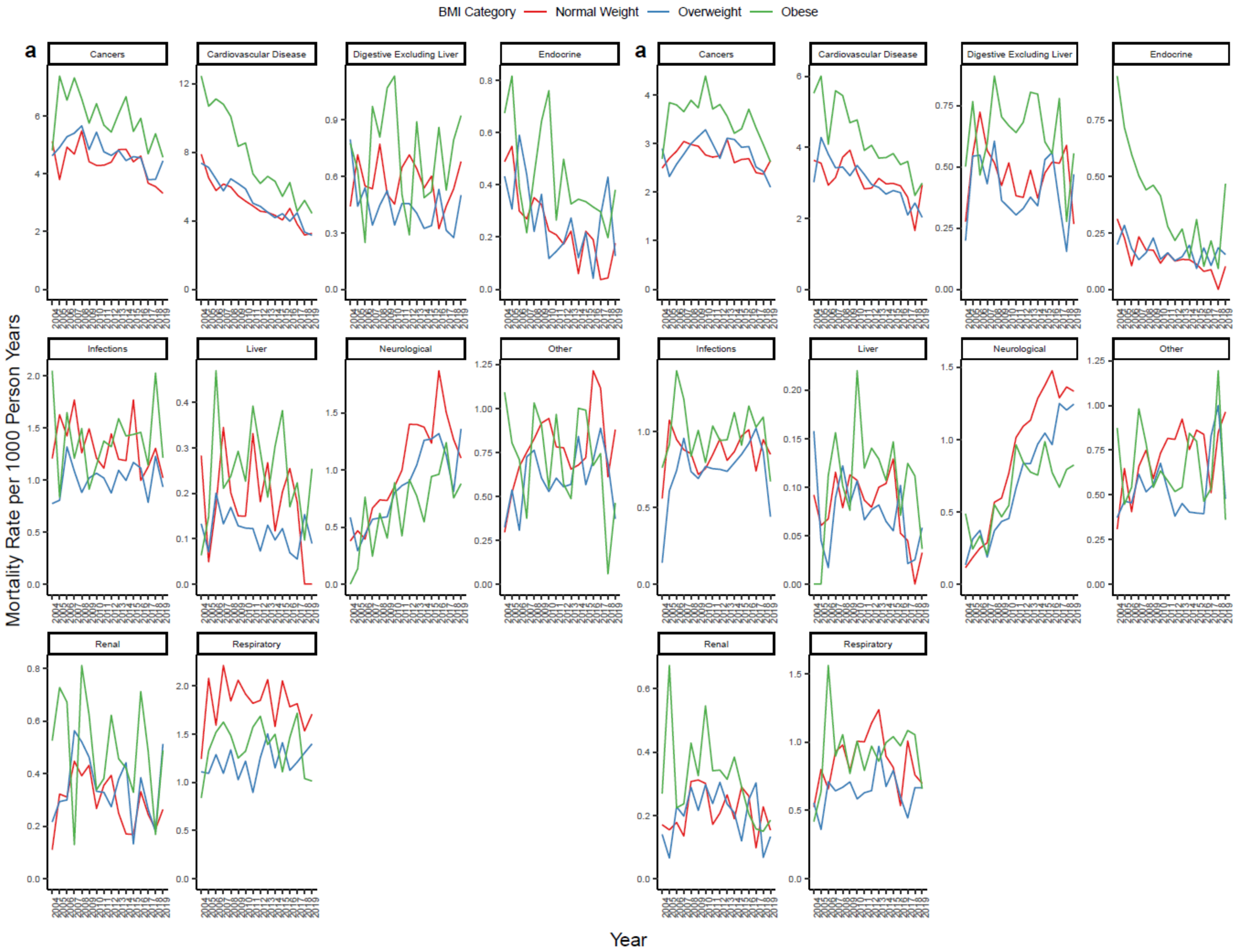

Appendix Figure 3. Age-adjusted mortality rates in Tier 3 outcomes between 2004 and 2019 in (a) males and (b) females

Appendix Table 3. Changes in proportional mortality (percent) for Tier 3 outcomes in 2004 and 2019 per 1000 person years and cumulative change in death rate across BMIs, by sex

	Normal Weight		Overweight		Obese	
	**2004 proportion of deaths**	**2019 proportion of deaths**	**2004 proportion of deaths**	**2019 proportion of deaths**	**2004 proportion of deaths**	**2019 proportion of deaths**
**MALES**						
**Tier 2**						
Non-cancer, non-cardiovascular disease NCDs	20·5 (19·5, 21·6)	36·8 (34·9, 38·7)	20·0 (19, 21·1)	34·3 (32·5, 36·1)	17·9 (16·9, 18·8)	28·5 (27, 29·9)
Cardiovascular disease	45·2 (42·8, 47·5)	26·3 (24·9, 27·7)	45·0 (42·7, 47·3)	24·5 (23·2, 25·7)	53·5 (50·7, 56·2)	30·3 (28·8, 31·9)
Cancer	29·4 (27·8, 30·9)	26·8 (25·4, 28·1)	28·3 (26·8, 29·7)	34·5 (32·7, 36·3)	20·7 (19·6, 21·7)	31·3 (29·6, 32·9)
Other	4·9 (4·7, 5·2)	10·1 (9·6, 10·6)	6·7 (6·4, 7·1)	6·8 (6·4, 7·1)	8·0 (7·6, 8·4)	9·9 (9·4, 10·5)
**Tier 3**						
Cancers	29·4 (27·8, 30·9)	26·8 (25·4, 28·1)	28·3 (26·8, 29·7)	34·5 (32·7, 36·3)	20·7 (19·6, 21·7)	31·3 (29·6, 32·9)
Cardiovascular disease	45·2 (42·8, 47·5)	26·3 (24·9, 27·7)	45 (42·7, 47·3)	24·5 (23·2, 25·7)	53·5 (50·7, 56·2)	30·3 (28·8, 31·9)
Digestive excluding liver	2·5 (2·4, 2·7)	5·5 (5·2, 5·8)	4·9 (4·6, 5·1)	3·9 (3·7, 4·1)	3·3 (3·2, 3·5)	6·3 (6·0, 6·6)
Endocrine	2·8 (2·7, 2·9)	1·4 (1·3, 1·5)	2·6 (2·5, 2·8)	1·0 (0·90, 1·0)	2·9 (2·7, 3)	2·6 (2·5, 2·7)
Infections	6·9 (6·6, 7·3)	8·2 (7·8, 8·7)	4·7 (4·5, 5·0)	7·2 (6·9, 7·6)	8·8 (8·4, 9·3)	8·3 (7·9, 8·7)
Liver	1·6 (1·5, 1·7)	0 (0, 0)	0·80 (0·80, 0·90)	0·70 (0·70, 0·70)	0·30 (0·30, 0·30)	1·7 (1·6, 1·8)
Neurological	2·2 (2·1, 2·3)	8·9 (8·4, 9·4)	3·6 (3·4, 3·8)	10·5 (10, 11·1)	0 (0, 0)	6·0 (5·7, 6·3)
Other	1·7 (1·6, 1·8)	7·1 (6·7, 7·4)	2·0 (1·9, 2·1)	2·9 (2·7, 3·0)	4·7 (4·4, 4·9)	3·2 (3·0, 3·3)
Renal	0·60 (0·60, 0·70)	2·1 (2·0, 2·2)	1·3 (1·3, 1·4)	4·0 (3·8, 4·2)	2·3 (2·1, 2·4)	3·3 (3·2, 3·5)
Respiratory	7·1 (6·8, 7·5)	13·7 (13, 14·4)	6·8 (6·4, 7·1)	10·8 (10·3, 11·4)	3·6 (3·4, 3·8)	6·9 (6·6, 7·3)
**FEMALES**						
**Tier 2**						
Non-cancer, non-cardiovascular disease NCDs	22·5 (20·9, 24)	37·3 (34·8, 39·9)	18·5 (17·2, 19·7)	38·0 (35·4, 40·6)	28·3 (26·4, 30·2)	31·5 (29·3, 33·6)
Cardiovascular disease	42·8 (39·9, 45·7)	29·5 (27·5, 31·5)	38·7 (36·0, 41·3)	26·1 (24·3, 27·9)	44·3 (41·3, 47·3)	31·9 (29·7, 34·1)
Cancers	29·4 (27·4, 31·4)	26·5 (24·7, 28·3)	37 (34·5, 39·6)	27·0 (25·2, 28·9)	21·5 (20·1, 23·0)	28·0 (26·1, 29·9)
Other	5·3 (5·0, 5·7)	6·7 (6·2, 7·2)	5·8 (5·4, 6·2)	8·9 (8·3, 9·5)	5·8 (5·4, 6·2)	8·6 (8·0, 9·2)
**Tier 3**						
Cancers	29·4 (27·4, 31·4)	26·5 (24·7, 28·3)	37·0 (34·5, 39·6)	27·0 (25·2, 28·9)	21·5 (20·1, 23)	28·0 (26·1, 29·9)
Cardiovascular disease	42·8 (39·9, 45·7)	29·5 (27·5, 31·5)	38·7 (36, 41·3)	26·1 (24·3, 27·9)	44·3 (41·3, 47·3)	31·9 (29·7, 34·1)
Digestive excluding liver	3·3 (3, 3·5)	2·7 (2·5, 2·9)	2·5 (2·4, 2·7)	6·0 (5·6, 6·5)	4·0 (3·7, 4·3)	5·9 (5·5, 6·3)
Endocrine	3·7 (3·4, 3·9)	1·0 (1·0, 1·1)	2·5 (2·4, 2·7)	2·0 (1·9, 2·1)	7·6 (7·1, 8·1)	5·0 (4·7, 5·3)
Infections	6·6 (6·2, 7·1)	8·5 (7·9, 9·1)	1·8 (1·7, 1·9)	5·7 (5·3, 6·1)	6·1 (5·7, 6·5)	7·2 (6·7, 7·7)
Liver	1·1 (1, 1·2)	0·30 (0·30, 0·30)	2 (1·9, 2·2)	0·70 (0·70, 0·80)	0 (0, 0)	0·40 (0·40, 0·40)
Neurological	1·3 (1·2, 1·4)	13·3 (12·4, 14·3)	1·7 (1·6, 1·8)	16·0 (14·9, 17·1)	3·9 (3·6, 4·2)	8·8 (8·2, 9·4)
Other	3·6 (3·4, 3·9)	9·6 (9·0, 10·3)	4·8 (4·4, 5·1)	6·1 (5·7, 6·6)	7·0 (6·5, 7·5)	3·8 (3·6, 4·1)
Renal	2·0 (1·9, 2·1)	1·5 (1·4, 1·6)	1·8 (1·7, 1·9)	1·7 (1·6, 1·8)	2·2 (2·0, 2·3)	2·0 (1·8, 2·1)
Respiratory	6·2 (5·8, 6·6)	7·0 (6·5, 7·5)	7·1 (6·6, 7·6)	8·6 (8, 9·1)	3·4 (3·1, 3·6)	7·0 (6·6, 7·5)

NCD - Non-communicable disease

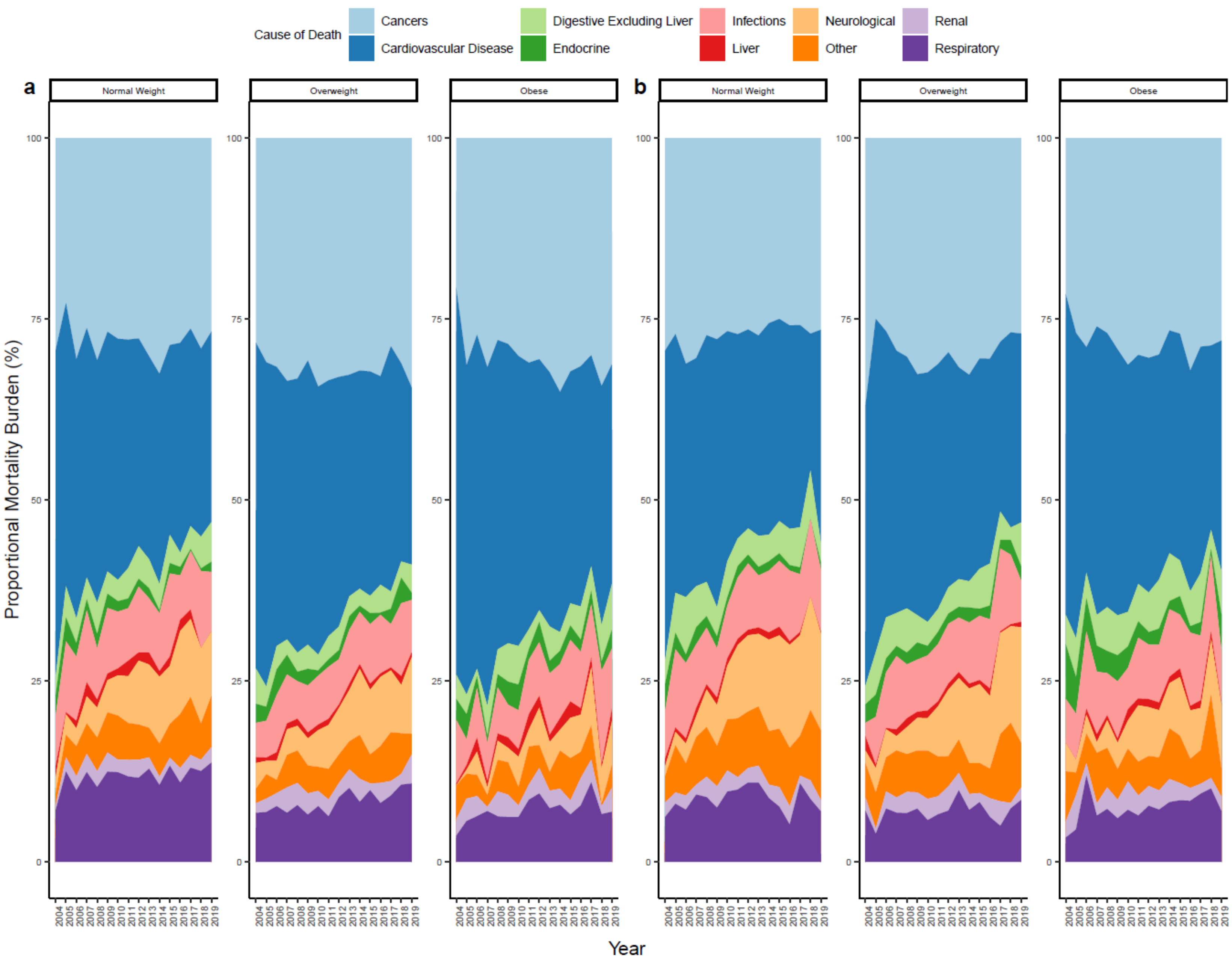

Appendix Figure 4. Proportional composition of Tier 3 outcomes between 2004 and 2019 in (a) males and (b) females

Appendix Table 4. Tier 3 outcomes and the proportional contribution (%) of mortality rankings in 2004 and 2019 by BMI in males

Normal Weight			
2004		2019	
Cardiovascular disease	45·2 (42·8, 47·5)	Cancers	26·8 (25·4, 28·1)
Cancers	29·4 (27·8, 30·9)	Cardiovascular disease	26·3 (24·9, 27·7)
Respiratory	7·1 (6·8, 7·5)	Respiratory	13·7 (13·0, 14·4)
Infections	6·9 (6·6, 7·3)	Neurological	8·9 (8·4, 9·4)
Endocrine	2·8 (2·7, 2·9)	Infections	8·2 (7·8, 8·7)
Digestive excluding liver	2·5 (2·4, 2·7)	Other	7·1 (6·7, 7·4)
Neurological	2·2 (2·1, 2·3)	Digestive excluding liver	5·5 (5·2, 5·8)
Other	1·7 (1·6, 1·8)	Renal	2·1 (2·0, 2·2)
Liver	1·6 (1·5, 1·7)	Endocrine	1·4 (1·3, 1·5)
Renal	0·60 (0·60, 0·70)	Liver	0 (0, 0)

Overweight			
2004		2019	
Cardiovascular disease	45 (42·7, 47·3)	Cancers	34·5 (32·7, 36·3)
Cancers	28·3 (26·8, 29·7)	Cardiovascular disease	24·5 (23·2, 25·7)
Respiratory	6·8 (6·4, 7·1)	Respiratory	10·8 (10·3, 11·4)
Digestive excluding liver	4·9 (4·6, 5·1)	Neurological	10·5 (10, 11·1)
Infections	4·7 (4·5, 5·0)	Infections	7·2 (6·9, 7·6)
Neurological	3·6 (3·4, 3·8)	Renal	4·0 (3·8, 4·2)
Endocrine	2·6 (2·5, 2·8)	Digestive excluding liver	3·9 (3·7, 4·1)
Other	2·0 (1·9, 2·1)	Other	2·9 (2·7, 3·0)
Renal	1·3 (1·3, 1·4)	Endocrine	1·0 (0·9, 1·0)
Liver	0·80 (0·80, 0·90)	Liver	0·70 (0·70, 0·70)

Obese			
2004		2019	
Cardiovascular disease	53·5 (50·7, 56·2)	Cancers	31·3 (29·6, 32·9)
Cancers	20·7 (19·6, 21·7)	Cardiovascular disease	30·3 (28·8, 31·9)
Infections	8·8 (8·4, 9·3)	Infections	8·3 (7·9, 8·7)
Other	4·7 (4·4, 4·9)	Respiratory	6·9 (6·6, 7·3)
Respiratory	3·6 (3·4, 3·8)	Digestive excluding liver	6·3 (6·0, 6·6)
Digestive excluding liver	3·3 (3·2, 3·5)	Neurological	6·0 (5·7, 6·3)
Endocrine	2·9 (2·7, 3·0)	Renal	3·3 (3·2, 3·5)
Renal	2·3 (2·1, 2·4)	Other	3·2 (3·0, 3·3)
Liver	0·30 (0·30, 0·30)	Endocrine	2·6 (2·5, 2·7)
Neurological	0 (0, 0)	Liver	1·7 (1·6, 1·8)

Appendix Table 5. Tier 3 outcomes and the proportional contribution (%) of mortality rankings in 2004 and 2019 by BMI in females

Normal Weight			
2004		2019	
Cardiovascular disease	42·8 (39·9, 45·7)	Cardiovascular disease	29·5 (27·5, 31·5)
Cancers	29·4 (27·4, 31·4)	Cancers	26·5 (24·7, 28·3)
Infections	6·6 (6·2, 7·1)	Neurological	13·3 (12·4, 14·3)
Respiratory	6·2 (5·8, 6·6)	Other	9·6 (9·0, 10·3)
Endocrine	3·7 (3·4, 3·9)	Infections	8·5 (7·9, 9·1)
Other	3·6 (3·4, 3·9)	Respiratory	7·0 (6·5, 7·5)
Digestive excluding liver	3·3 (3, 3·5)	Digestive excluding liver	2·7 (2·5, 2·9)
Renal	2·0 (1·9, 2·1)	Renal	1·5 (1·4, 1·6)
Neurological	1·3 (1·2, 1·4)	Endocrine	1·0 (1·0, 1·1)
Liver	1·1 (1·0, 1·2)	Liver	0·30 (0·30, 0·30)

Overweight			
2004		2019	
Cardiovascular disease	38·7 (36·0, 41·3)	Cancers	27·0 (25·2, 28·9)
Cancers	37·0 (34·5, 39·6)	Cardiovascular disease	26·1 (24·3, 27·9)
Respiratory	7·1 (6·6, 7·6)	Neurological	16·0 (14·9, 17·1)
Other	4·8 (4·4, 5·1)	Respiratory	8·6 (8·0, 9·1)
Digestive excluding liver	2·5 (2·4, 2·7)	Other	6·1 (5·7, 6·6)
Endocrine	2·5 (2·4, 2·7)	Digestive excluding liver	6·0 (5·6, 6·5)
Liver	2·0 (1·9, 2·2)	Infections	5·7 (5·3, 6·1)
Infections	1·8 (1·7, 1·9)	Endocrine	2·0 (1·9, 2·1)
Renal	1·8 (1·7, 1·9)	Renal	1·7 (1·6, 1·8)
Neurological	1·7 (1·6, 1·8)	Liver	0·70 (0·70, 0·80)

Obese			
2004		2019	
Cardiovascular disease	44·3 (41·3, 47·3)	Cardiovascular disease	31·9 (29·7, 34·1)
Cancers	21·5 (20·1, 23)	Cancers	28·0 (26·1, 29·9)
Endocrine	7·6 (7·1, 8·1)	Neurological	8·8 (8·2, 9·4)
Other	7·0 (6·5, 7·5)	Infections	7·2 (6·7, 7·7)
Infections	6·1 (5·7, 6·5)	Respiratory	7·0 (6·6, 7·5)
Digestive excluding liver	4·0 (3·7, 4·3)	Digestive excluding liver	5·9 (5·5, 6·3)
Neurological	3·9 (3·6, 4·2)	Endocrine	5·0 (4·7, 5·3)
Respiratory	3·4 (3·1, 3·6)	Other	3·8 (3·6, 4·1)
Renal	2·2 (2, 2·3)	Renal	2·0 (1·8, 2·1)
Liver	0 (0, 0)	Liver	0·40 (0·40, 0·40)

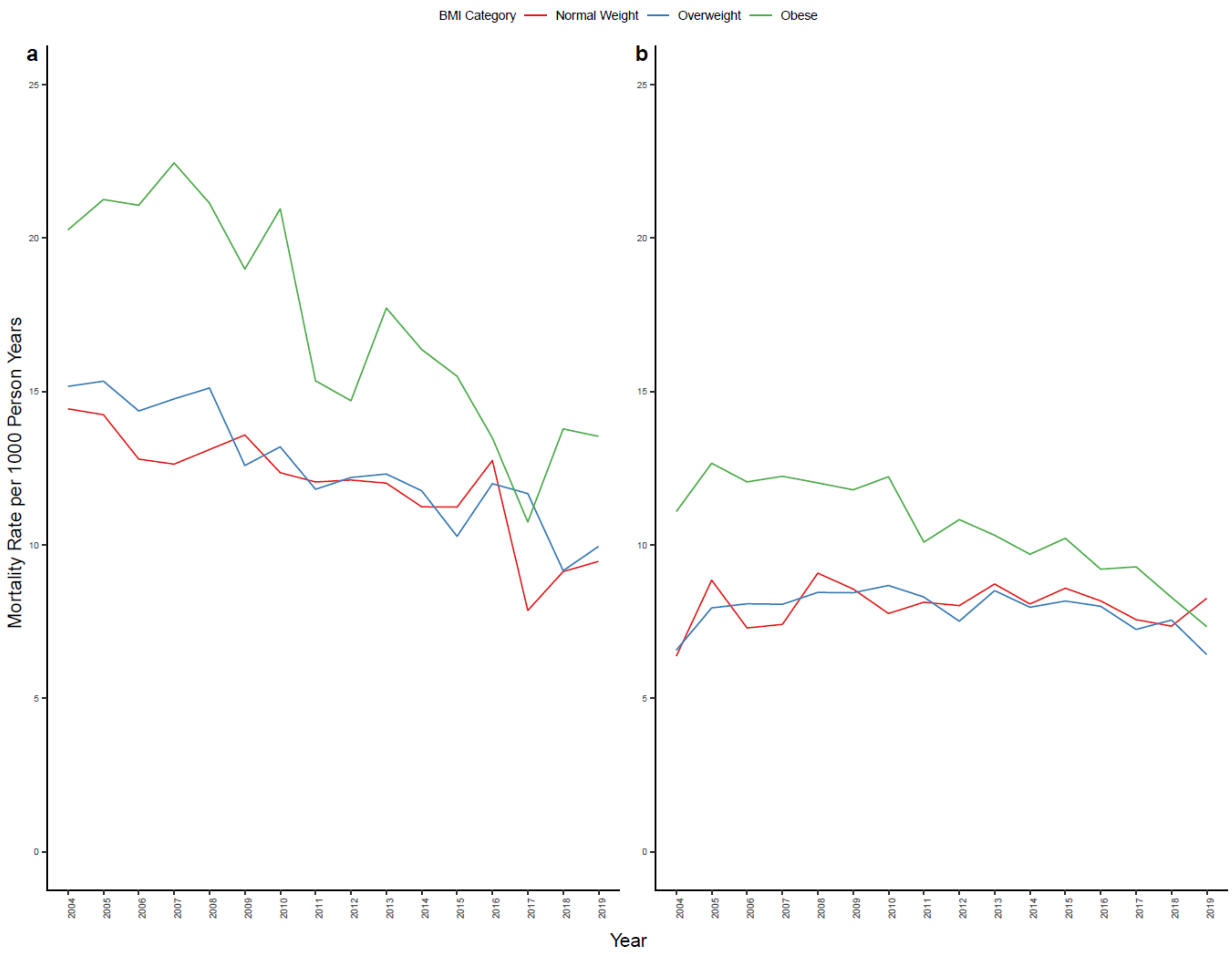

Appendix Figure 5. Age-adjusted all-cause mortality rates in never smokers between 2004 and 2019 in (a) males and (b) females

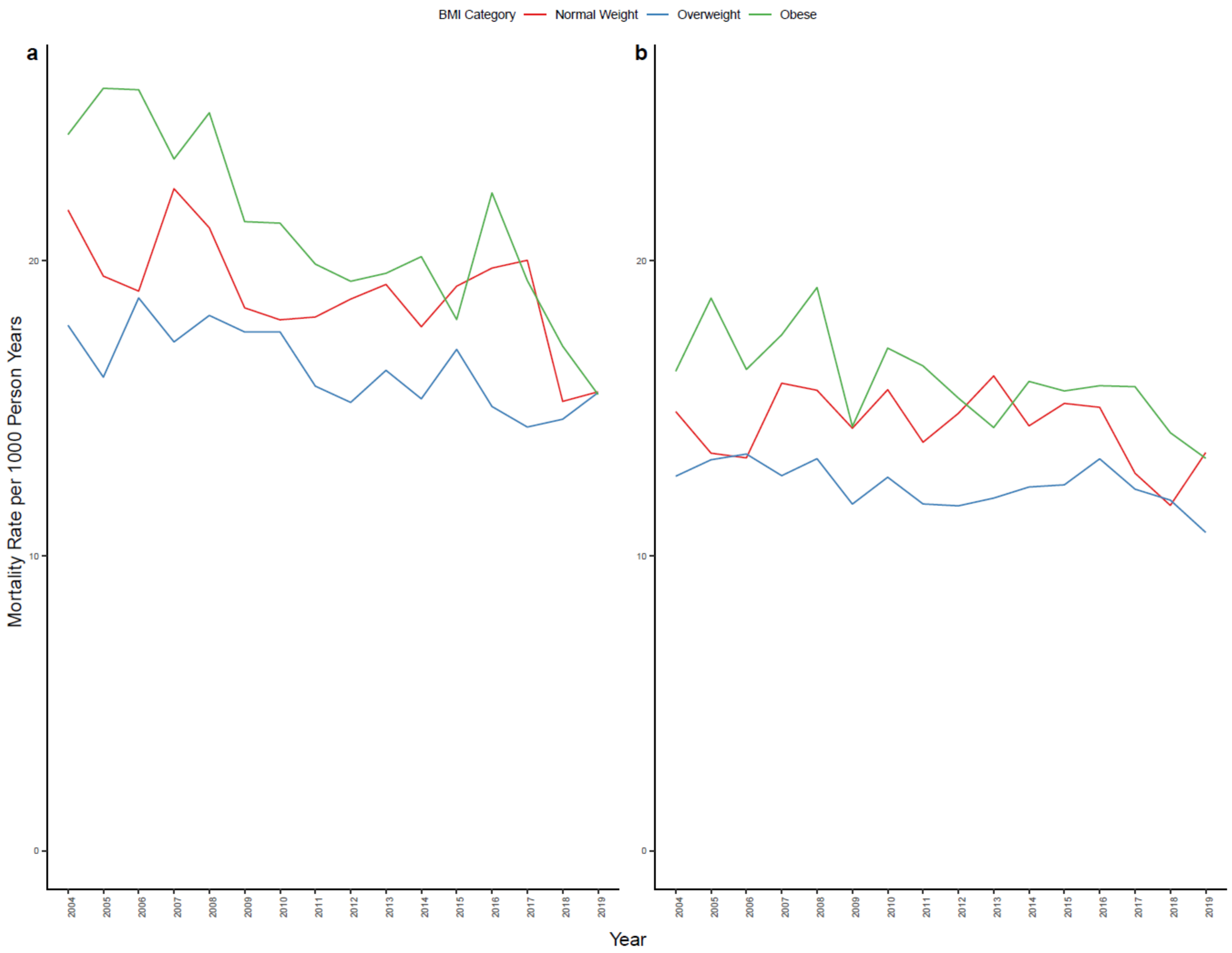

Appendix Figure 6. Age-adjusted all-cause mortality rates in current/ex-smokers between 2004 and 2019 in (a) males and (b) females

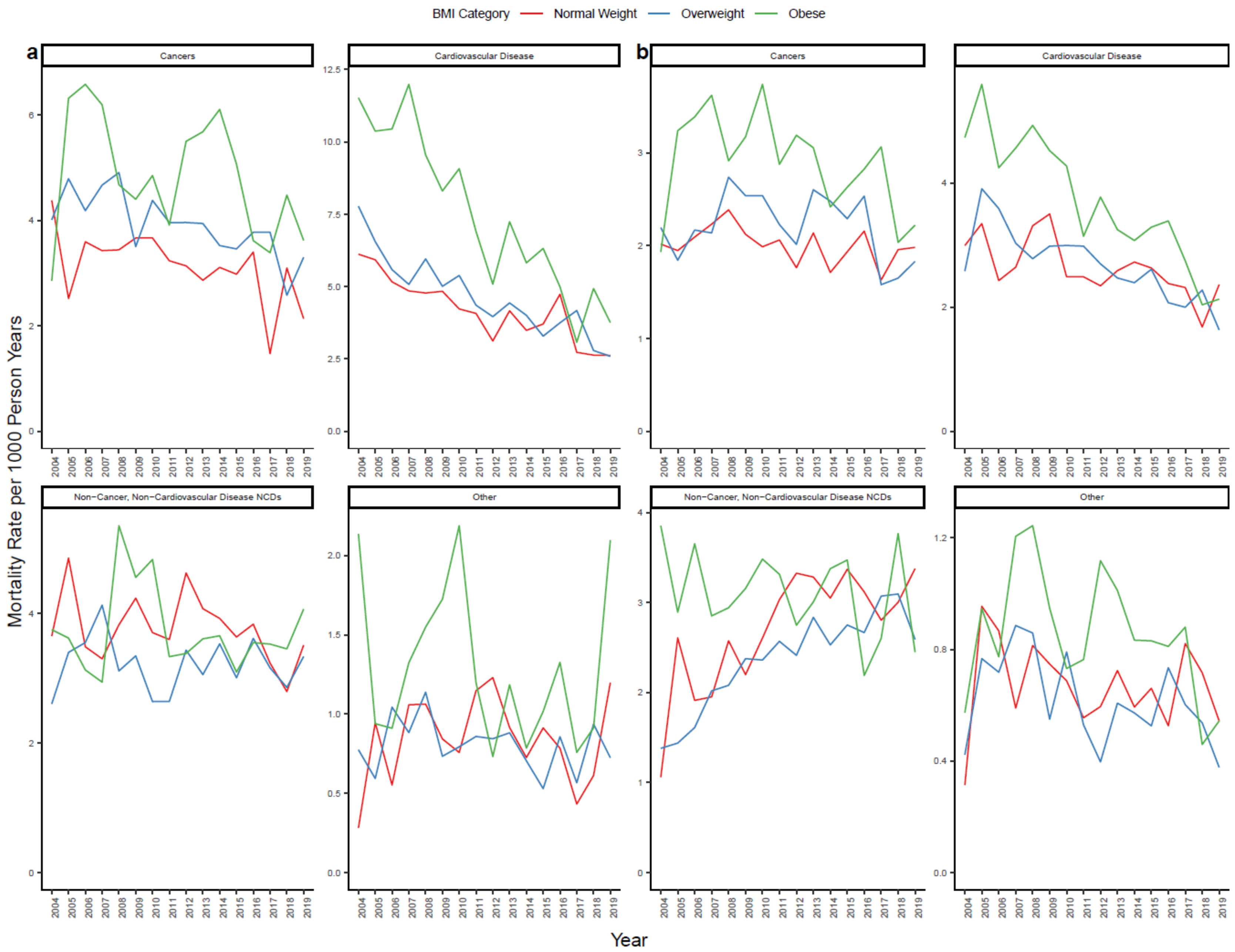

Appendix Figure 7. Age-adjusted mortality rates in never smokers across Tier 2 outcomes between 2004 and 2019 in (a) males and (b) females

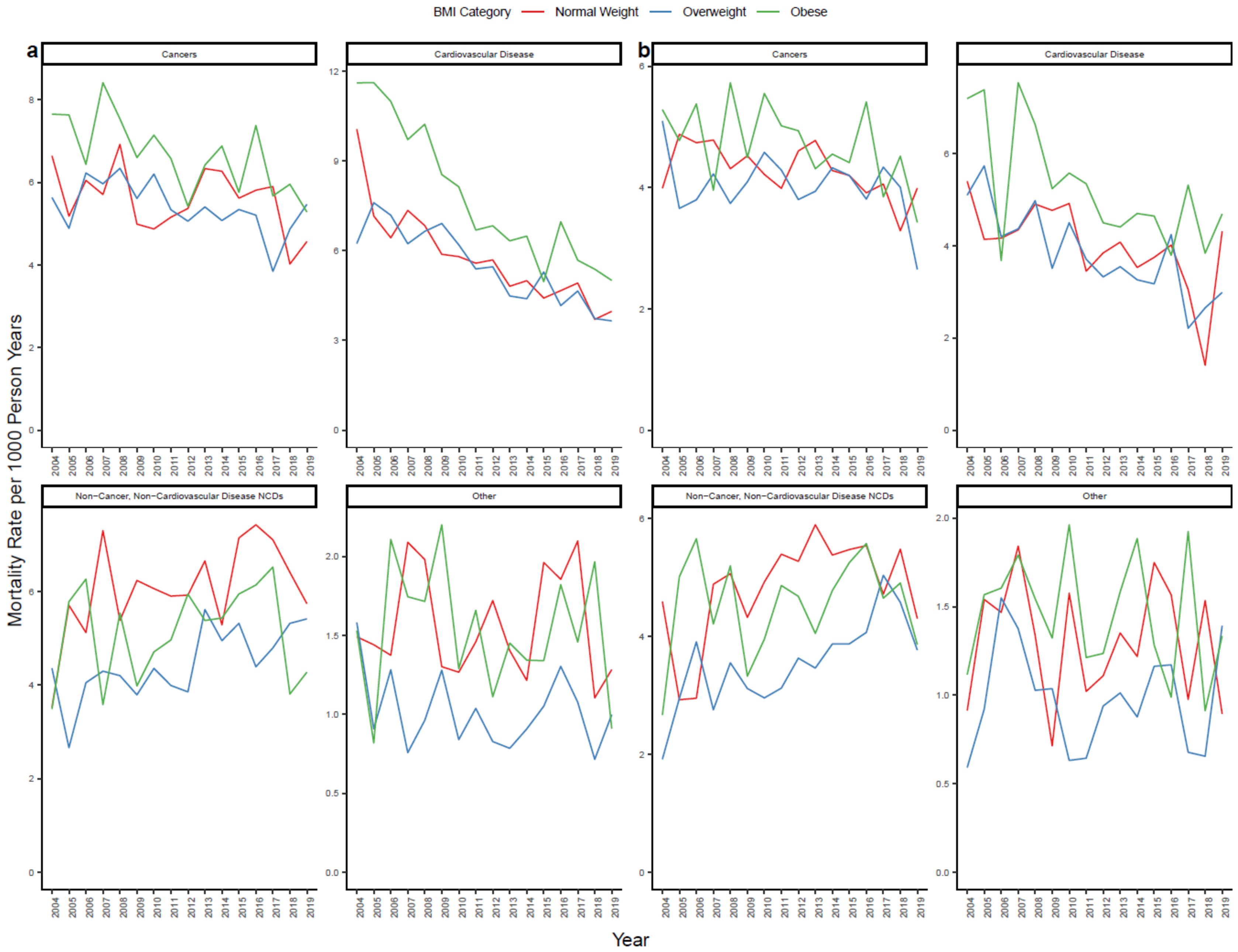

Appendix Figure 8. Age-adjusted mortality rates in current/ex-smokers across Tier 2 outcomes between 2004 and 2019 in (a) males and (b) females

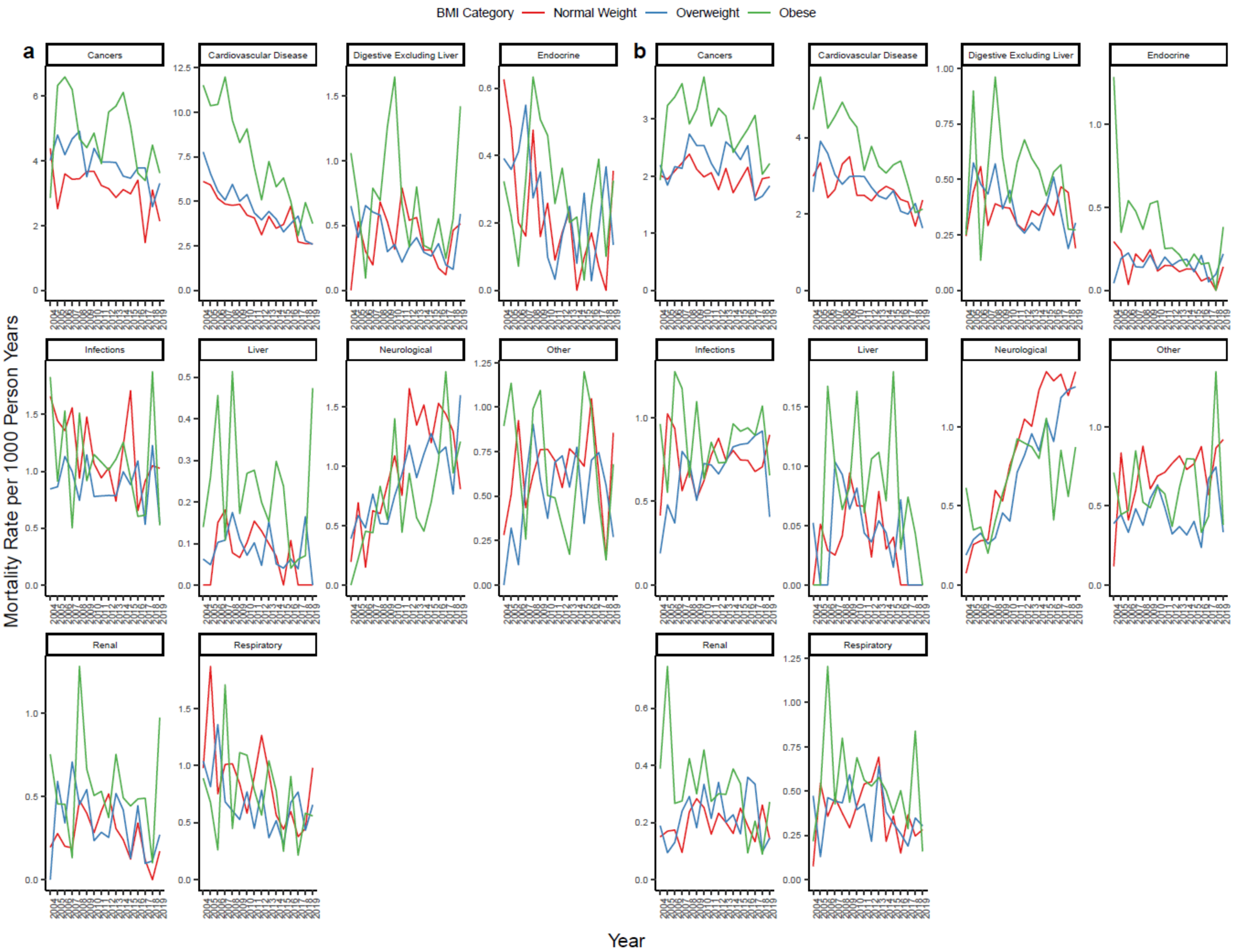

Appendix Figure 9. Age-adjusted mortality rates in never smokers across Tier 3 outcomes between 2004 and 2019 in (a) males and (b) females

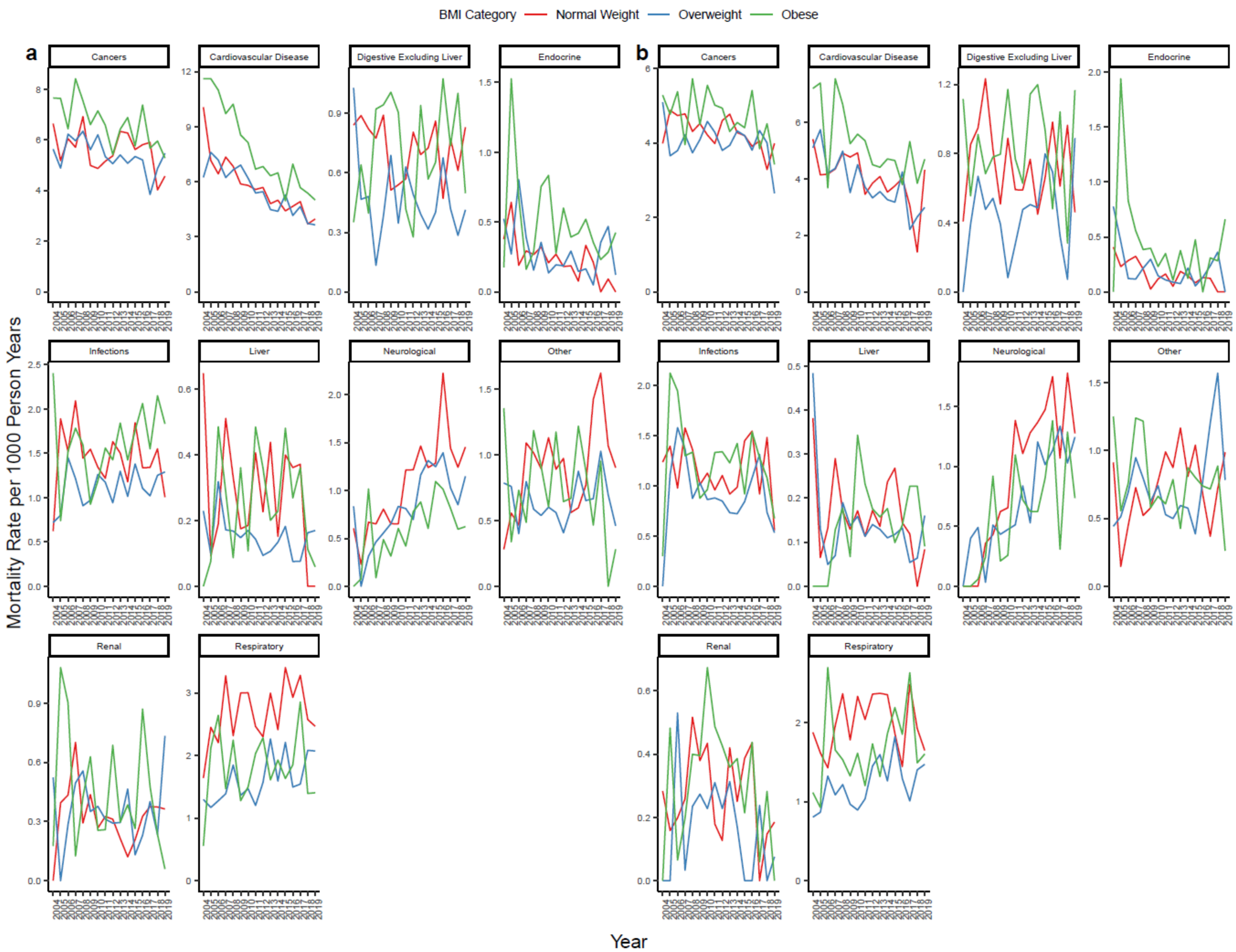

Appendix Figure 10. Age-adjusted mortality rates in current/ex-smokers across Tier 3 outcomes between 2004 and 2019 in (a) males and (b) females

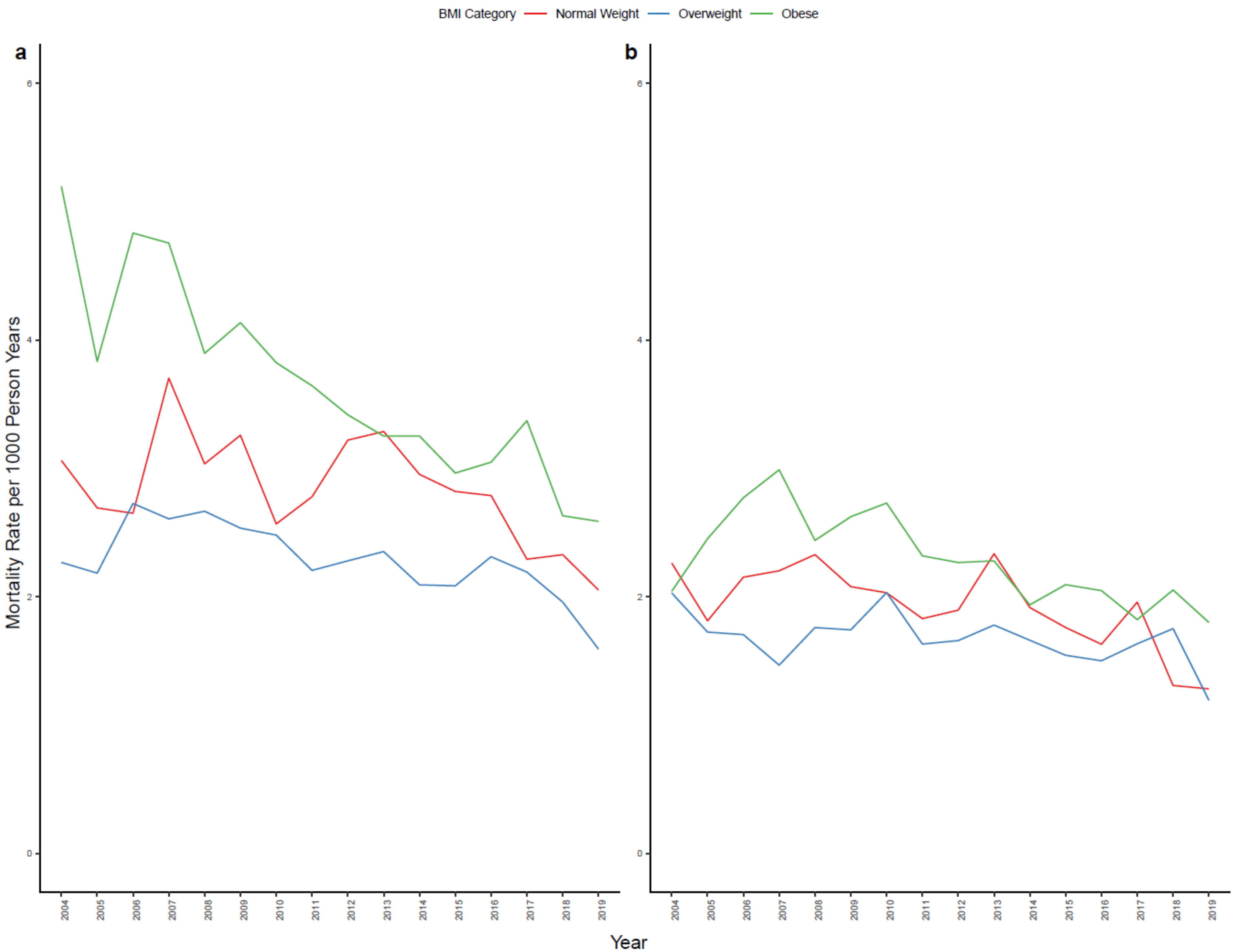

Appendix Figure 11. Age-adjusted all-cause mortality rates in 35-74 year olds between 2004 and 2019 in (a) males and (b) females

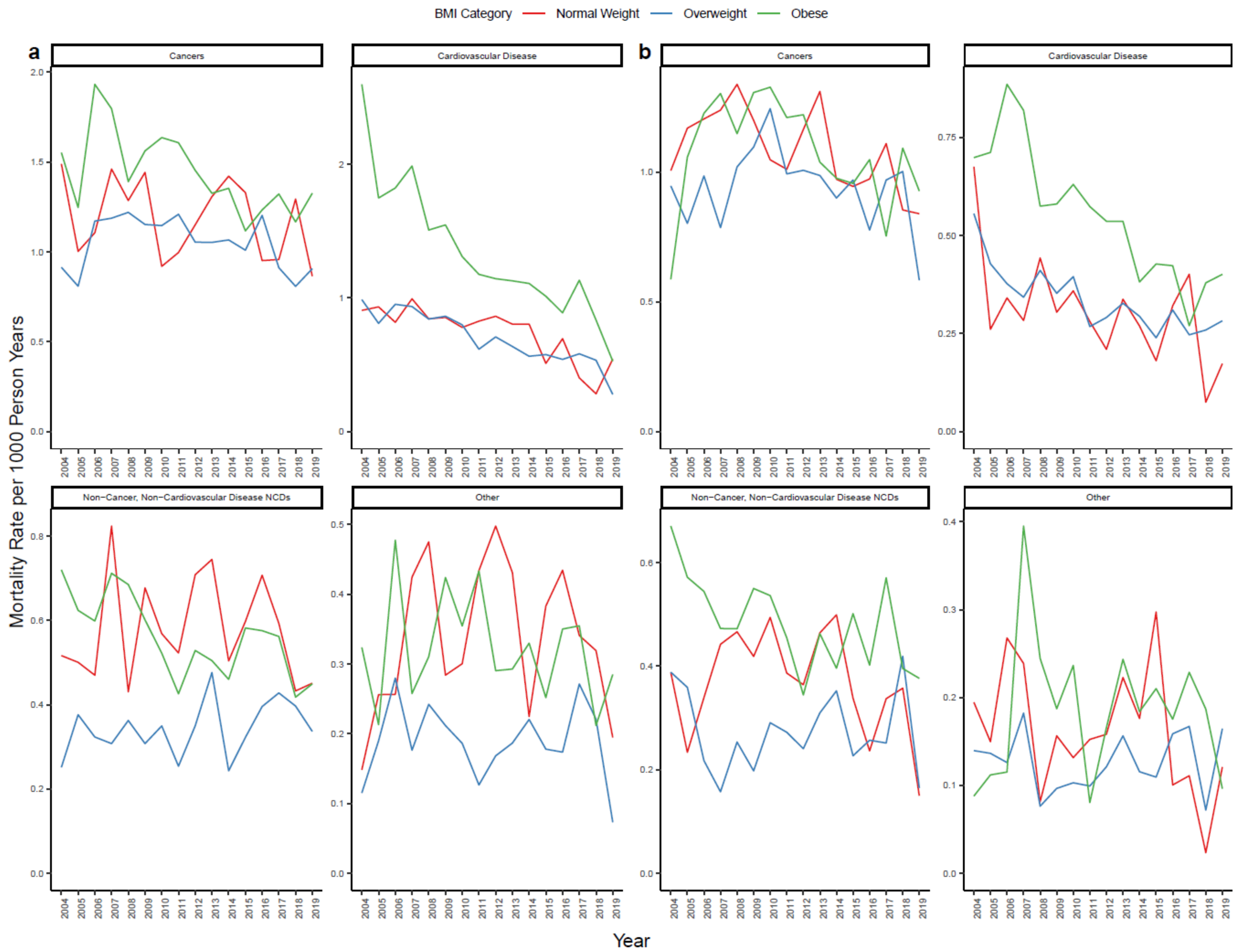

Appendix Figure 12. Age-adjusted mortality rates in 35-74 year olds across Tier 2 outcomes between 2004 and 2019 in (a) males and (b) females

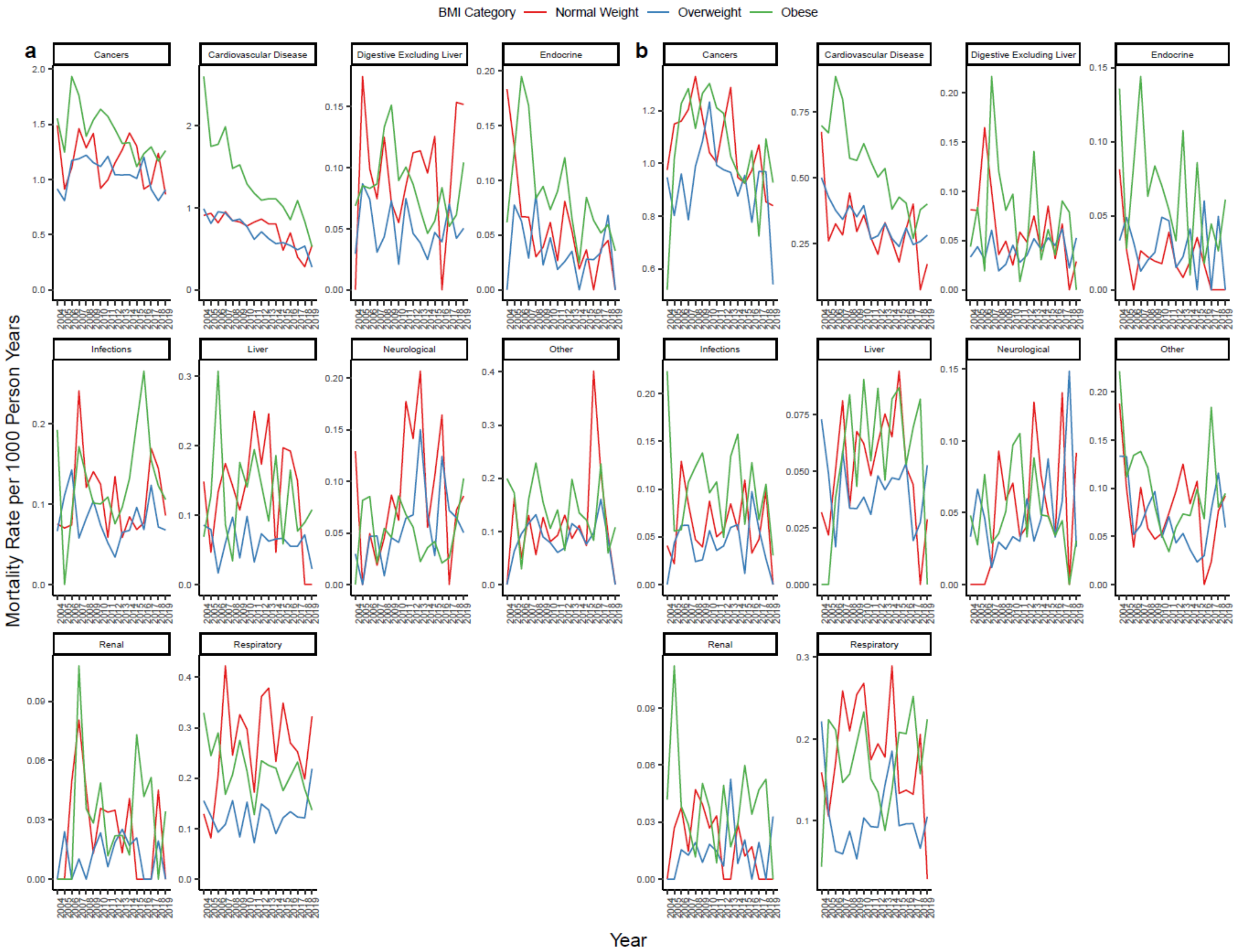

Appendix Figure 13. Age-adjusted mortality rates in 35-74 year olds across Tier 3 outcomes between 2004 and 2019 in (a) males and (b) females

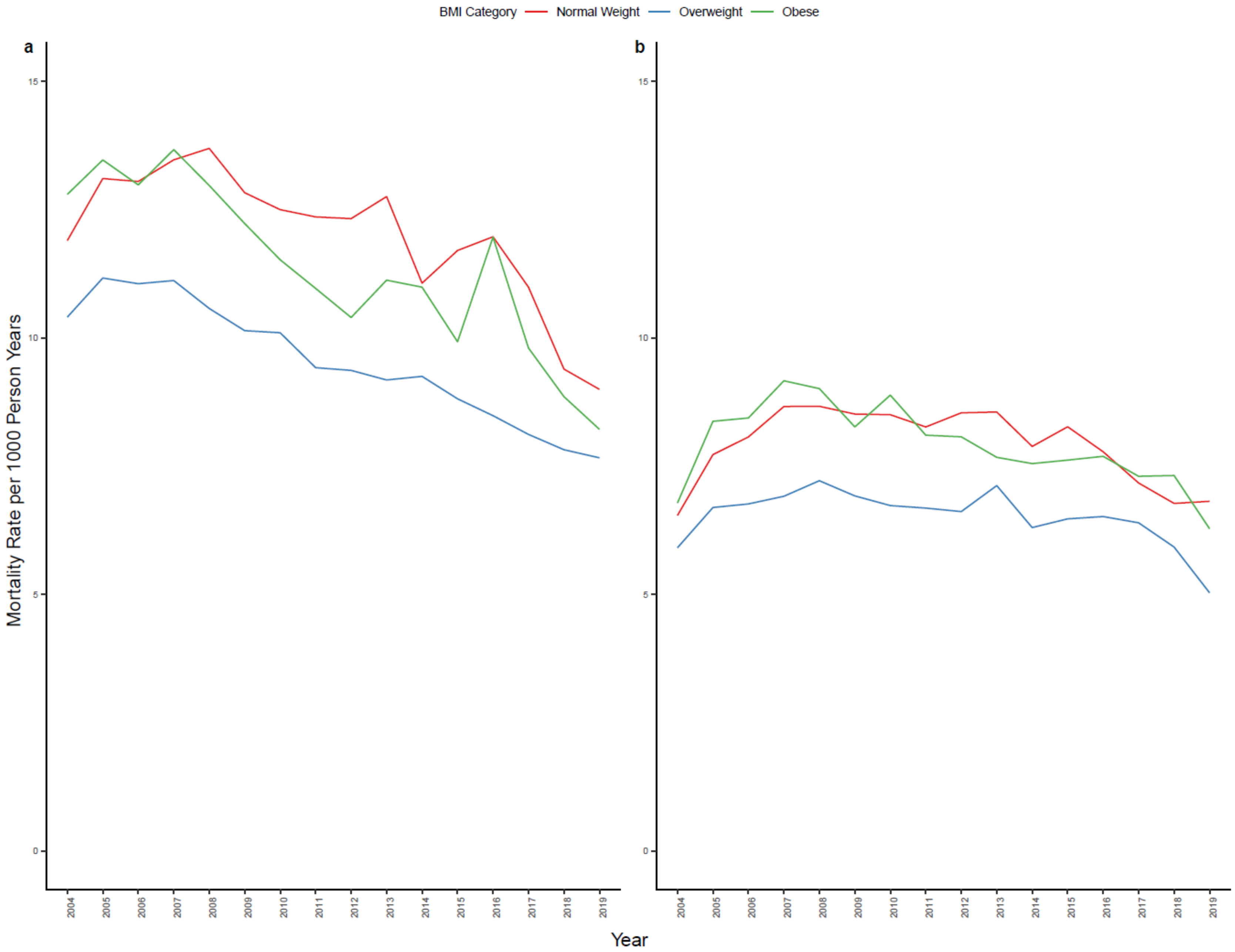

Appendix Figure 14. Sensitivity analysis with a two-year exclusion period: age-adjusted mortality rates in Tier 1 outcomes in (a) males and (b) females

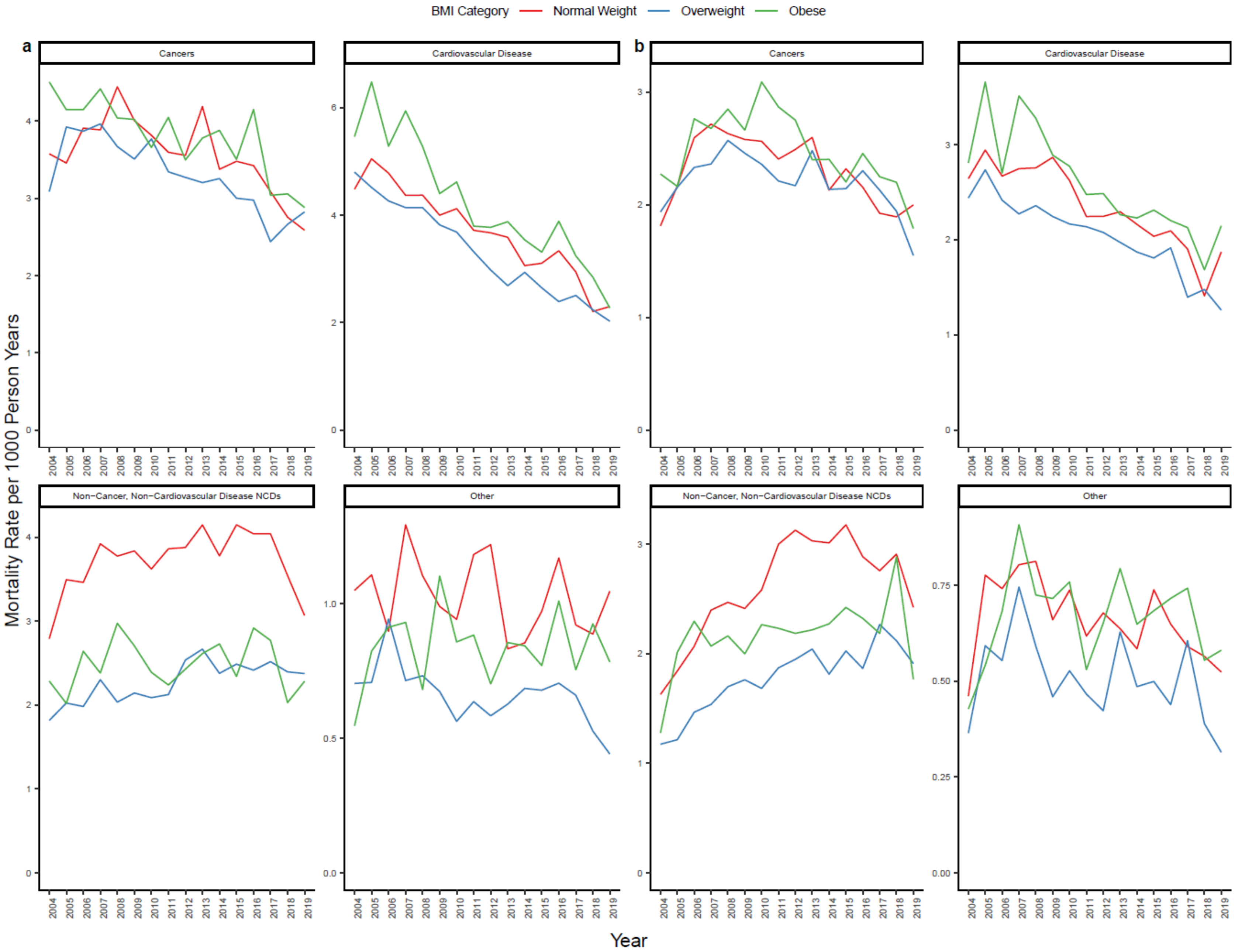

Appendix Figure 15. Sensitivity analysis with a two-year exclusion period: age-adjusted mortality rates in Tier 2 outcomes in (a) males and (b) females

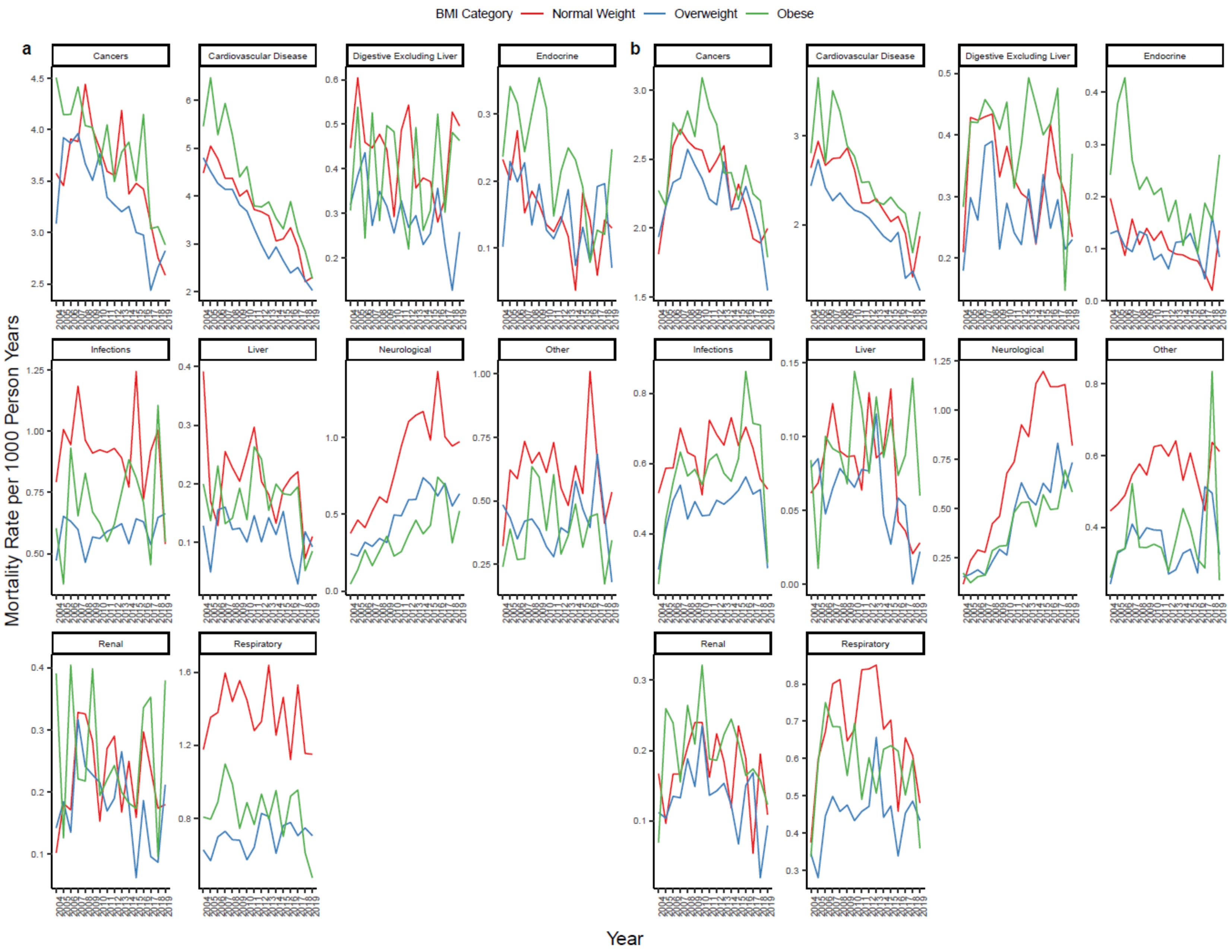

Appendix Figure 16. Sensitivity analysis with a two-year exclusion period: age-adjusted mortality rates in Tier 3 outcomes in (a) males and (b) females
